# Development and validation of an explainable machine learning-based risk prediction model for obesity in Chinese children and adolescents: a population-based study

**DOI:** 10.3389/fnut.2026.1816724

**Published:** 2026-05-05

**Authors:** Zekai Chen, Lin Zhu, Peijie Chen

**Affiliations:** 1 School of Exercise and Health, Shanghai University of Sport, Shanghai, China; 2 School of Sport and Health, Guangzhou Sport University, Guangzhou, China; 3 Innovative Research Center for Sports Science in the Guangdong-Hong Kong-Macao Greater Bay Area, Guangzhou Sport University, Guangzhou, China

**Keywords:** children and adolescents, machine learning, obesity, prediction model, SHAP

## Abstract

**Background:**

Childhood obesity represents a significant global public health challenge. Accurate and rapid prediction models for identifying obesity risk in children and adolescents are essential for facilitating early prevention and enabling timely interventions. However, interpretable and user-friendly obesity risk prediction models based on nationally representative data remain limited. This study aimed to develop and validate a model to predict current obesity risk among children and adolescents in China.

**Methods:**

The models were developed using cross-sectional data from the 2017–2018 Physical Activity and Fitness in China—The Youth Study (PAFCTYS; *n* = 35,016) and were temporally validated with 2020 data (*n* = 3,495). Candidate predictors (*n* = 38), primarily encompassing physical activity, sedentary behavior, and sociodemographic variables, were measured concurrently with the outcome. The participants included individuals from 31 administrative regions across China. Features were identified using the Least Absolute Shrinkage and Selection Operator (LASSO) with recursive feature elimination (RFE), and predictive models were developed using eight different machine learning algorithms. The model’s performance was evaluated using metrics such as AUC, sensitivity, specificity, Matthews Correlation Coefficient (MCC), and Brier score. The model was interpreted using the SHapley Additive exPlanation (SHAP) method.

**Results:**

The random forest (RF) model outperformed all other models, achieving an AUC of 1.000 on the training set and 0.946 on the testing set. It also maintained good discriminative performance on the temporal validation dataset, with an AUC of 0.810. The RF model also shows the highest accuracy, specificity, and MCC, and the lowest Brier score. SHAP analysis indicates that parental BMI, using mobile electronic devices on weekdays, MVPA (moderate-to-vigorous physical activity), watching TV on weekdays, and sex are the top 5 most important features in the obesity risk prediction model. A web-based risk calculator based on the RF model has been successfully deployed.

**Conclusion:**

Using a large, nationally representative sample and readily available variables, we successfully developed and validated an obesity risk prediction model for Chinese children and adolescents using explainable machine learning techniques. The model can quickly and accurately assess an individual’s current obesity risk, enabling healthcare agencies, schools, and parents to conduct large-scale obesity risk screening.

## Introduction

Most countries have already failed to meet the WHO’s 2025 obesity target, which aimed to prevent any increase in obesity rates between 2010 and 2025 (1). According to a study by the NCD Risk Factor Collaboration, in 2022, 159 million school-aged children and adolescents were obese, with obesity rates of 9.3% for boys and 6.9% for girls (2). A recent study estimated that if no action is taken, by 2050, there will be 360 million children and adolescents globally suffering from obesity (3). This indicates that the escalating trend of obesity in youth remains inadequately addressed, necessitating intensified efforts toward its prevention and management. Previous investigations have largely focused on weight loss interventions for overweight and obese youth (4–6). While these treatment-oriented approaches are necessary, they inadequately address upstream prevention of obesity onset. Given the persistent rise in obesity rates among children and adolescents, there is a critical need to shift from treatment-oriented strategies to prevention-oriented approaches that enable early identification of individuals at high risk of obesity. In 2024, the Chinese government introduced the Technical Guidelines for Comprehensive Public Health Work on Overweight and Obesity in Primary and Secondary Schools, detailing a three-tier prevention strategy emphasizing prevention, early intervention, and disease warning (7). However, the successful implementation of such prevention-oriented policies requires tools that are highly accurate and practical for individual-level risk screening and stratification. In recent years, predictive modeling has emerged as a promising tool for identifying high-risk children and adolescents with obesity. However, the vast majority of existing prediction models for childhood obesity have significant limitations. Firstly, most studies involve participants from a single center or region, lacking national representativeness and limiting the generalizability (8–11). Second, although some studies have developed prediction models, their primary purpose was to identify factors influencing obesity, and they have not achieved quantitative risk stratification for individual obesity risk (12–15). Furthermore, in terms of utility, several existing models incorporate feature variables that require specialized laboratory assessments, sophisticated equipment, or high recall difficulty (e.g., specific biomarkers, physical fitness test, or birth weight), which substantially increases application costs and operational complexity, thereby limiting their feasibility for large-scale obesity risk screening (16–19). Moreover, machine learning algorithms frequently pose interpretability challenges, often referred to as the “black-box” issue. To address the black-box problem of machine learning (ML) algorithms, recent studies have integrated the SHapley Additive exPlanation (SHAP) technique with predictive modeling to improve interpretability (20, 21). However, few predictive models of childhood and adolescent obesity employ interpretable algorithms. Given the gaps above, there is an urgent need to develop a nationally representative, easily accessible, and highly interpretable predictive model to assess obesity risk in children and adolescents.

In this study, we aimed to develop and validate an explainable machine learning model to identify current obesity risk among Chinese children and adolescents using a nationally representative cohort. This study has several innovations, including the use of nationally representative sample data and readily obtainable variables for developing models using various machine learning algorithms and for performing temporal validation. Additionally, we performed an interpretability analysis of the model and deployed it as a mobile web application, enabling large-scale, rapid screening and providing a practical foundation for addressing the implementation gap between national prevention policies and frontline practice.

## Methods

### Study design

This study employed a cross-sectional design to develop predictive models for identifying current obesity risk among children and adolescents. The data for this study were drawn from the 2017–2018 Physical Activity and Fitness in China—The Youth Study (PAFCTYS). The PAFCTYS was a national survey aimed at assessing the physical fitness and health of Chinese children and adolescents. This comprehensive cross-sectional study was carried out across 31 administrative regions in China. To achieve balanced representation of economic development and rural–urban diversity, administrative cities and districts/towns were selected through stratification by socioeconomic status within urban and rural categories. Details regarding the design and methodologies employed in PAFCTYS can be found in the published articles (22–24). The Ethics Review Board at Shanghai University of Sport approved this study. Given that the research posed negligible risk to the participants, and taking into account the scale and cultural context of the studied populations, the Committee decided to waive the requirement for written consent and granted approval for the use of verbal consent. The Ethics Review Committee approved a verbal consent protocol mandating researchers to secure authorization from school educators and administrators before starting data collection. This included detailing the study’s objectives, possible risks, and benefits of participating.

### Participants

To ensure the representativeness of the research sample, data sampling strictly adhered to the fundamental principles of regional, school-level, and sex balance. The sample size of boys and girls from elementary, middle, and high schools was determined using sex and grade distribution data from the Ministry of Education of the People’s Republic of China. Student demographics by province, educational level, and sex in mainland China are available on the Ministry of Education’s official website. Finally, the sampling proportions for students across different school levels and sexes within each province were determined, and 35,016 participants were randomly selected from PAFCTYS’s database.

### Data collection and processing

In this study, data were collected through questionnaires and on-site measurements, and a total of 38 candidate variables were used as potential predictors to develop the prediction models. Among these variables, no outcome-related proxies were included as predictors (Supplementary Table S1). The questionnaire items used in this study have all been published and validated in previous research (24–31). For detailed information about the questionnaire items used in this study, please refer to the Supplementary material. The information from the questionnaires concerned both students and their parents. For students, the collected variables include: demographic information (age and sex), lifestyle behaviors (sleep duration, physical activity, sedentary behavior, and sport participation), exercise intention, and BMI. For parents, the collected data included lifestyle behaviors (sedentary behavior and physical activity), attitude toward exercise, and BMI. Details can be found in Supplementary Table S1.

The dataset was randomly split into training and testing sets in a 70:30 ratio for model training and testing, respectively. Then, outlier detection was performed. An outlier is identified as a value exceeding the upper quartile by more than 1.5 times the interquartile range, or falling below the lower quartile by the same margin. To maintain consistency with the original data distribution, these outliers were substituted with the corresponding boundary values. For missing data, we used the ‘mice’ package to perform multivariate imputation by chained equations (MICE). Data preprocessing was performed separately for the training, testing, and temporal validation sets to avoid data leakage. The MICE method was applied for multiple imputation on the training, testing, and temporal validation set. In the training set, 32 of the 38 candidate feature variables underwent imputation. In contrast, 8 feature variables were imputed in both the testing and temporal validation sets. The outcome variable was not included in the imputation process. The specific variables imputed in each dataset, along with their proportions of missing values, are presented in Supplementary Tables S9–S11.

To mitigate the data imbalance in the original dataset, which could affect the model’s predictive performance, we employed the Synthetic Minority Oversampling Technique (SMOTE) to balance the samples in addition to our analysis using the original imbalanced dataset (32). SMOTE was applied to the training set to balance the number of negative and positive outcome samples. To ensure a fair evaluation and prevent data leakage, SMOTE was applied only to the training set (33). The test set and temporal validation were left unbalanced.

### Feature selection

Initially, we applied the least absolute shrinkage and selection operator (LASSO) algorithm to select variables. LASSO can address multicollinearity and prevent overfitting by applying a penalty that shrinks variable coefficients (34, 35). Subsequently, we applied recursive feature elimination (RFE) to further select variables, enhancing the predictive model’s efficacy and robustness. RFE is a widely used feature selection technique in machine learning (36). This method operates by eliminating non-essential features in order to ultimately identify the most effective combination of features, thus enhancing the overall performance of the model. Both LASSO and RFE used 10-fold cross-validation and were applied only to the training set. To preserve the original distribution information during the feature selection stage and avoid bias introduced by synthetic data in feature selection algorithms, which could interfere with the identification of key features (37, 38), we did not repeat the feature screening process on the SMOTE-processed training set.

### Model development and evaluation

Eight machine learning algorithms were employed to predict obesity risk in children and adolescents: Logistic Regression (LR), Support Vector Machine (SVM), Gradient Boosting Machine (GBM), Neural Network (NNET), Random Forest (RF), eXtreme Gradient Boosting (XGBoost), K-Nearest Neighbor (KNN), and Adaptive Boosting (AdaBoost). We applied the above algorithms to build models on both the original imbalanced dataset and the SMOTE-processed balanced dataset. If the AUC of the optimal model on the balanced dataset was higher than that on the imbalanced dataset, we reported the final results based on the balanced dataset, otherwise, we reported the results from the original imbalanced dataset. The model was optimized using grid search with 10-fold cross-validation from the ‘caret’ package, alongside manual fine-tuning, to identify the optimal hyperparameters for each model. We assessed model reliability using indicators such as the area under the receiver operating characteristic curve (AUC), sensitivity, specificity, positive predictive value (PPV), negative predictive value (NPV), accuracy, F1 score, and Matthews correlation coefficient (MCC). Logarithmic Loss (Log-Loss) was used to measure the difference between a model’s predicted probability distribution and the actual labels. Furthermore, we constructed a calibration curve to evaluate the agreement between the model’s predicted probabilities and the actual outcomes. We utilized Decision Curve Analysis (DCA) to assess the model’s net benefit at different threshold levels. Finally, we determined the best model based on its performance across the aforementioned evaluation indicators and methods.

### Model explanation

SHAP is a game theory-based approach for interpreting machine learning models. This approach applies the Shapley value from cooperative game theory to assign a “contribution score” to each feature, reflecting its influence on the model’s predictions. This approach helps clarify the decision-making process of complex black-box models by quantifying the importance of individual features (39). The calculation of SHAP values is implemented through the “shapviz” package.

### Web calculator deployment

To enhance the model’s applicability, the prediction model has been integrated into a web platform via a Shiny application. The application can generate an obesity risk score for children and adolescents once the feature variable values associated with the finalized model are provided.

### Statistical analysis

**Figure 1:**
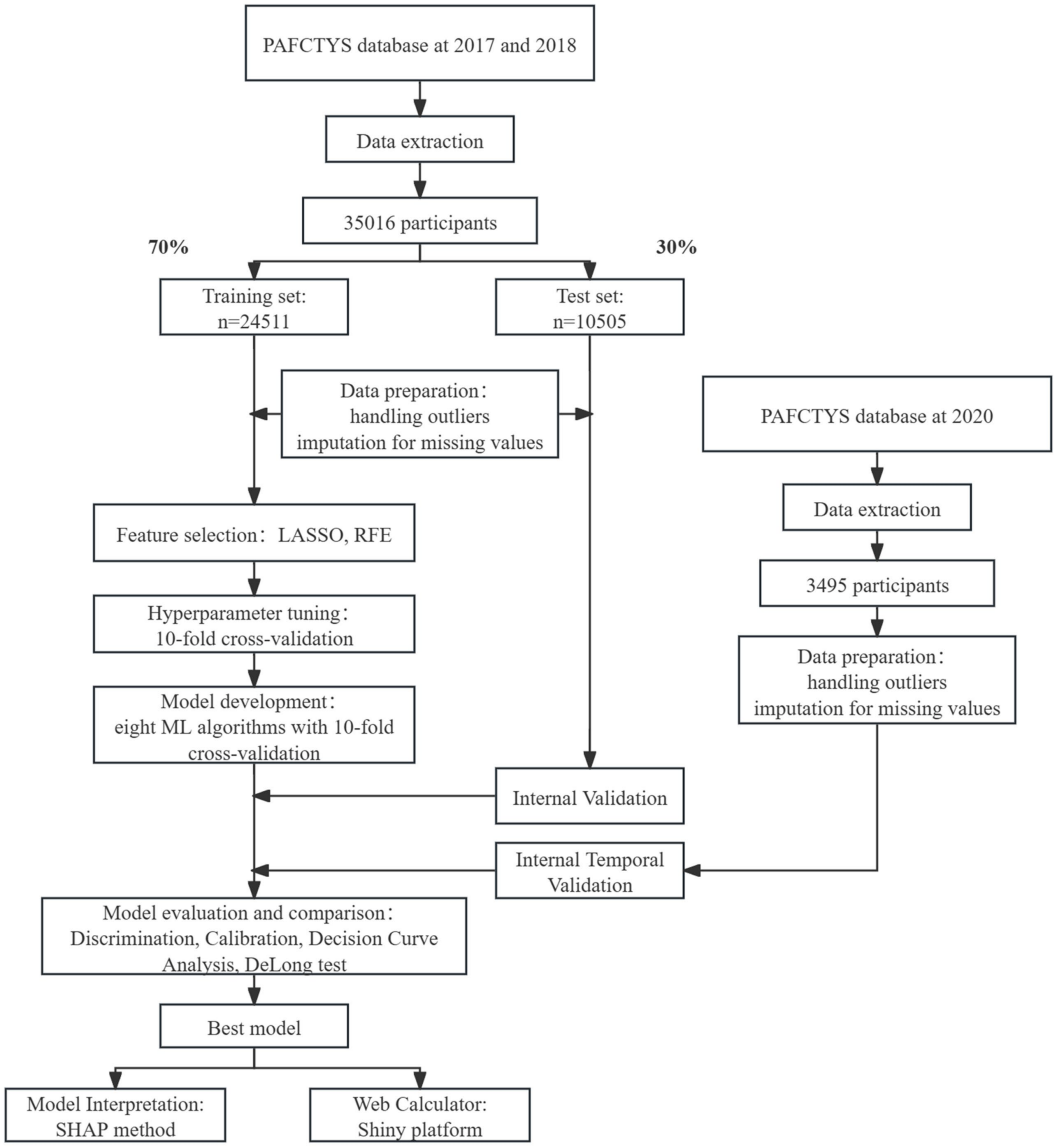
Flowchart of this study.

The workflow of this study is shown in Figure 1. Data analyses were performed using R (v4.4.2) and SPSS (v24.0). Continuous variables with a normal distribution are presented as mean ± standard deviation and evaluated using the t-test. Skewed continuous variables are presented as medians with interquartile ranges (IQR) and analyzed using the Mann–Whitney U test or the Kruskal–Wallis H test. Categorical variables are displayed as counts with percentages and are compared using the Chi-square test. Using DeLong’s test to compare the AUC of the predictive models to reflect the differences in performance between the two models. A *p*-value below 0.05 in a two-tailed test was considered statistically significant.

## Results

### Selection of predictor variables

**Figure 2:**
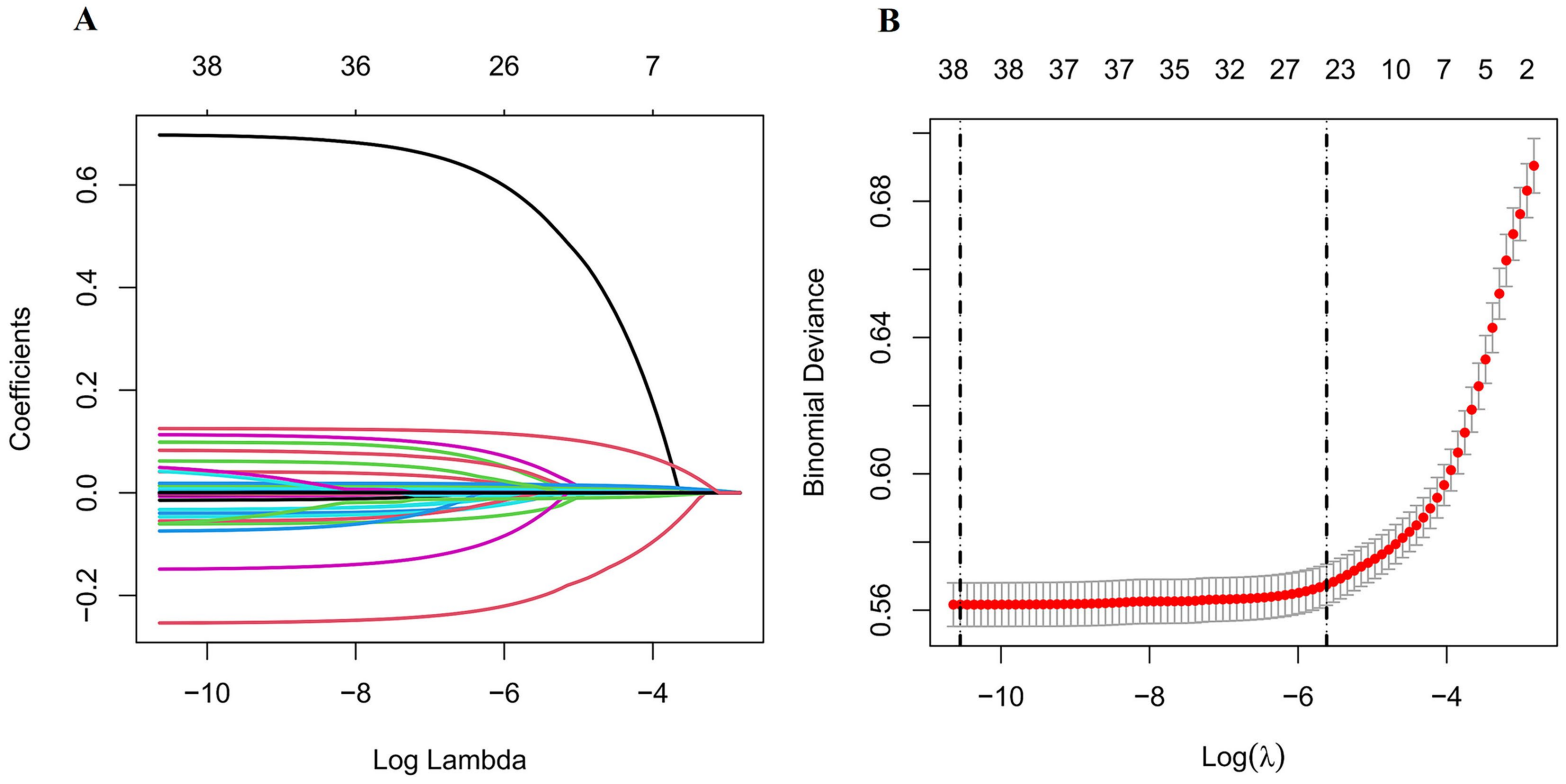
Feature selection based on LASSO regression. **(A)** A visual representation of the coefficient profiles derived from the logarithmic sequence of lambda values. **(B)** The selection of lambda using both the minimum criteria (illustrated by the left dotted line) and the 1-standard error criteria (represented by the right dotted line).

**Figure 3:**
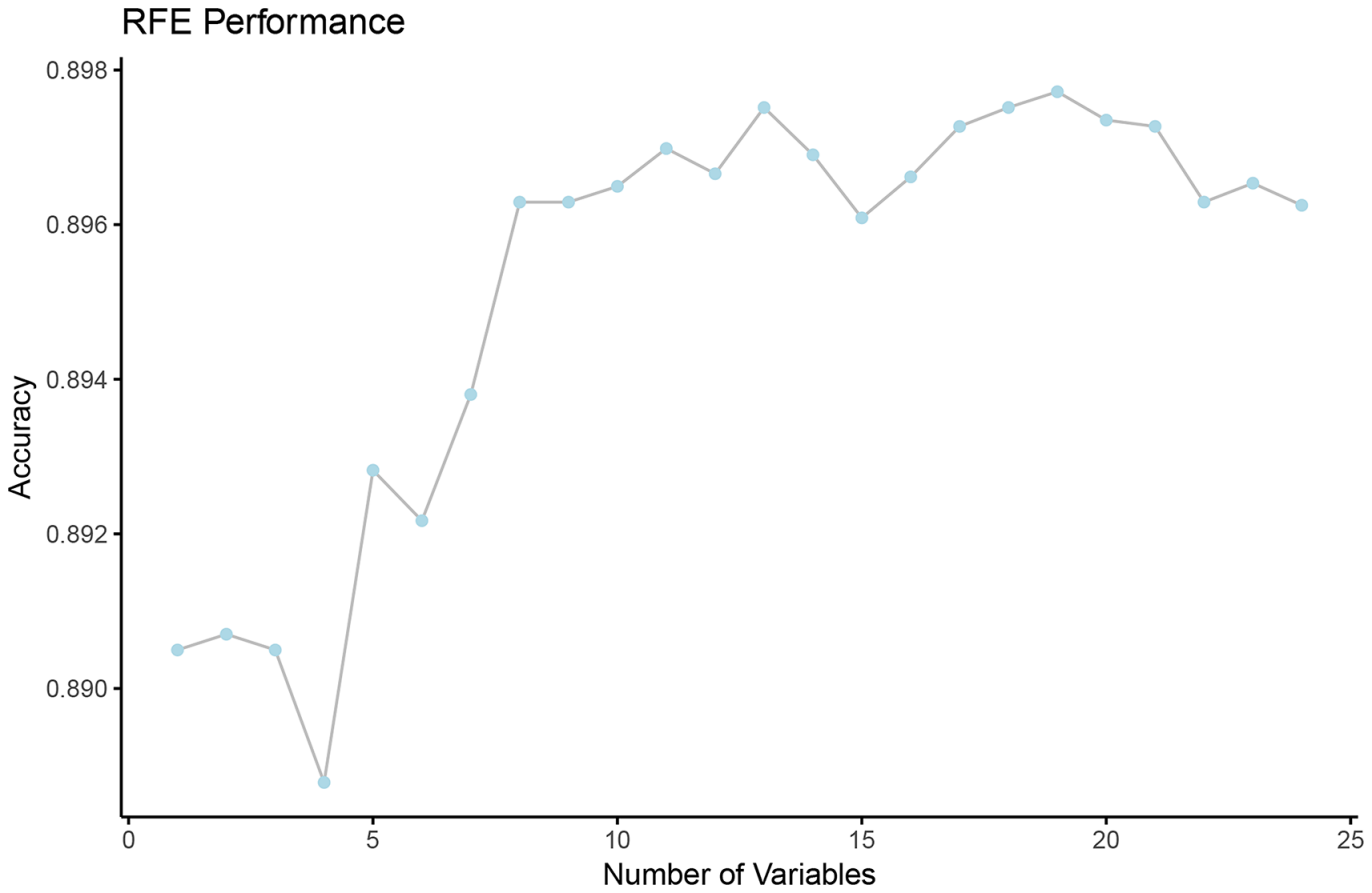
Feature selection process based on RFE. RFE, recursive feature elimination.

First, we employed the LASSO method for the initial selection of feature variables, identifying 24 feature variables based on the Lambda = 1se criterion (Figure 2). Then, we employed RFE to further filter the 24 feature variables obtained from LASSO, resulting in the optimal feature subset, as shown in Figure 3. The optimal feature subset includes 13 feature variables: MVPA, Parental BMI, VPA, using mobile electronic devices on weekdays, Watching TV on weekdays, Sex, Age, Using the computer on weekdays, Parental sedentary time on weekdays, Watching TV on weekends, Using mobile electronic devices on weekends, Exercise frequency in school sports teams, and Exercise frequency in external sports training classes. Due to the correlation coefficient r > 0.6 between MVPA and VPA, the final model includes the remaining 12 feature variables, excluding VPA.

### Model development

**Figure 4:**
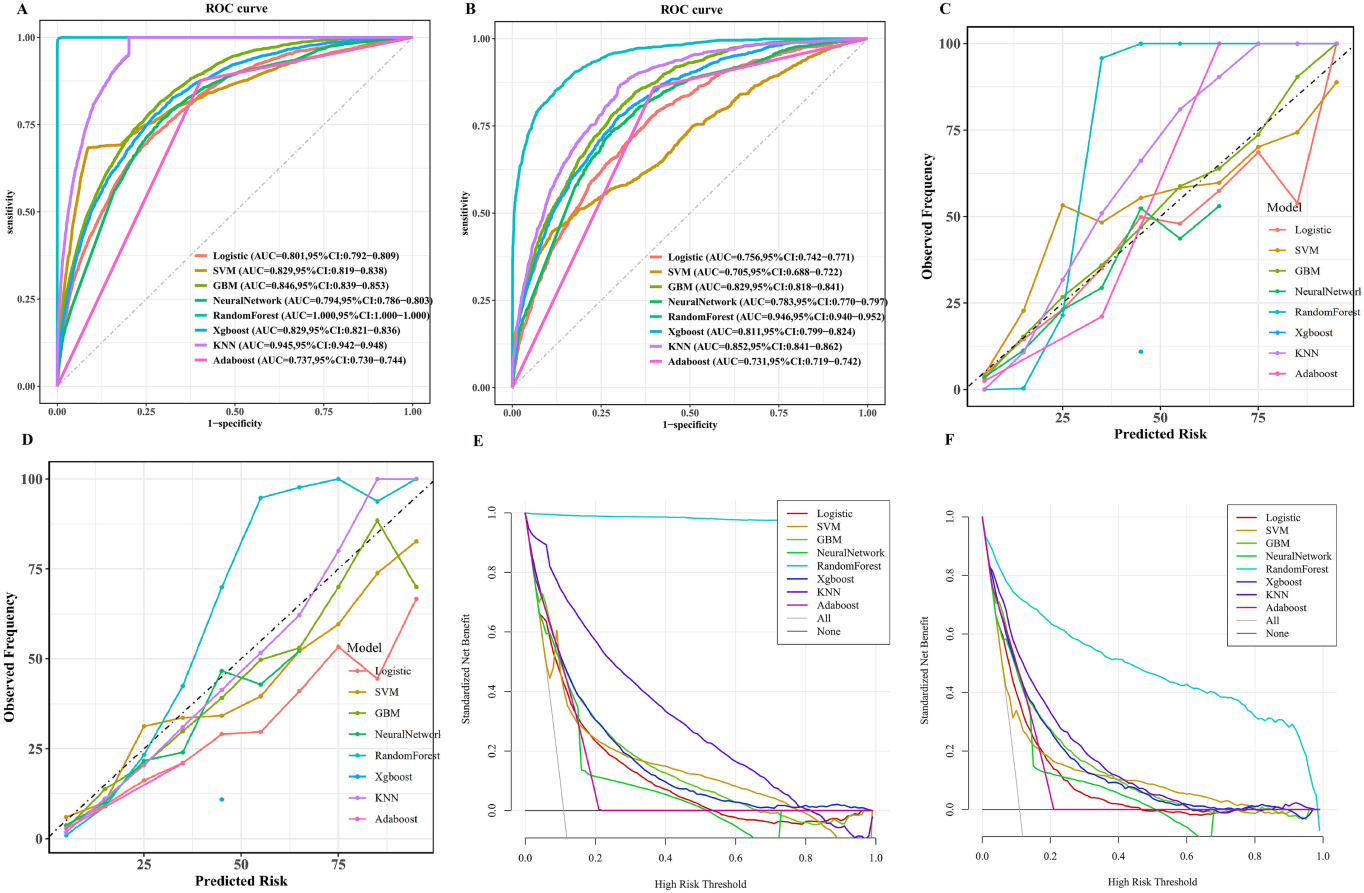
Evaluation of obesity risk prediction models for children and adolescents in both training and testing datasets ROC curve **(A,B)**; calibration curve **(C,D)**; and DCA curve **(E,F)**. SVM, Support Vector Machine; XGBoost, eXtreme Gradient Boosting; KNN, K-nearest Neighbors; GBM, Gradient Boosting Machine; AdaBoost, Adaptive Boosting.

As shown in Table 1 and Figures 4A,C, the RF model demonstrated excellent performance in both discrimination and calibration on the training set, with an AUC of 1 and a Brier score of 0.023. The RF model achieved the highest accuracy (0.996), specificity (0.966), precision (0.966), F1 score (0.981), and MCC (0.979). The RF model achieved the highest accuracy with a Log-Loss value of 0.0939 (Supplementary Table S4). The findings from DeLong’s test indicated that the RF model’s AUC was markedly superior to those of the alternative models (*p* < 0.001; Supplementary Table S5). The DCA results indicated that the RF model delivered a net benefit and demonstrated superior performance compared with alternative models across the entire risk threshold range of 0 to 100% (Figure 4E).

**Table 1:** The performance of machine learning models across training and testing datasets.

Model	Accuracy	Sensitivity	Specificity	Precision	F1 score	MCC	Brier score
Training set							
LR	0.728 (0.722, 0.734)	0.722 (0.704, 0.739)	0.728 (0.722, 0.734)	0.246 (0.237, 0.257)	0.367 (0.355, 0.380)	0.301 (0.288, 0.314)	0.083 (0.081, 0.085)
SVM	0.888 (0.885, 0.892)	0.684 (0.666, 0.703)	0.914 (0.910, 0.917)	0.493 (0.476, 0.509)	0.573 (0.557, 0.588)	0.519 (0.503, 0.535)	0.079 (0.076, 0.082)
GBM	0.722 (0.716, 0.727)	0.815 (0.801, 0.830)	0.710 (0.704, 0.716)	0.257 (0.248, 0.266)	0.391 (0.379, 0.402)	0.344 (0.333, 0.357)	0.077 (0.075, 0.079)
Neural Network	0.711 (0.705, 0.717)	0.766 (0.750, 0.782)	0.704 (0.698, 0.710)	0.242 (0.232, 0.251)	0.367 (0.356, 0.379)	0.308 (0.296, 0.321)	0.085 (0.083, 0.087)
RF	0.996 (0.995, 0.997)	0.997 (0.996, 0.999)	0.996 (0.995, 0.997)	0.966 (0.959, 0.973)	0.981 (0.978, 0.985)	0.979 (0.975, 0.983)	0.023 (0.019, 0.028)
XGBoost	0.731 (0.725, 0.736)	0.779 (0.763, 0.794)	0.725 (0.719, 0.731)	0.258 (0.248, 0.268)	0.388 (0.376, 0.399)	0.334 (0.322, 0.347)	0.221 (0.216, 0.225)
KNN	0.820 (0.816, 0.825)	1.000 (1.000, 1.000)	0.798 (0.794, 0.803)	0.379 (0.367, 0.391)	0.549 (0.537, 0.562)	0.550 (0.540, 0.560)	0.062 (0.060, 0.063)
AdaBoost	0.627 (0.621, 0.633)	0.877 (0.865, 0.889)	0.596 (0.589, 0.603)	0.211 (0.203, 0.219)	0.340 (0.330, 0.351)	0.297 (0.287, 0.308)	0.095 (0.093, 0.097)
Test set							
LR	0.628 (0.618, 0.637)	0.775 (0.750, 0.799)	0.610 (0.599, 0.619)	0.196 (0.185, 0.208)	0.313 (0.298, 0.329)	0.242 (0.224, 0.260)	0.095 (0.093, 0.098)
SVM	0.839 (0.831, 0.845)	0.449 (0.418, 0.477)	0.887 (0.880, 0.892)	0.327 (0.304, 0.349)	0.378 (0.355, 0.401)	0.293 (0.268, 0.318)	0.086 (0.082, 0.090)
GBM	0.675 (0.666, 0.684)	0.847 (0.826, 0.868)	0.654 (0.644, 0.663)	0.231 (0.219, 0.244)	0.363 (0.347, 0.380)	0.319 (0.303, 0.337)	0.080 (0.078, 0.082)
Neural Network	0.723 (0.714, 0.731)	0.733 (0.708, 0.758)	0.722 (0.712, 0.730)	0.245 (0.229, 0.260)	0.367 (0.348, 0.385)	0.302 (0.282, 0.322)	0.086 (0.083, 0.089)
RF	0.875 (0.869, 0.882)	0.851 (0.830, 0.872)	0.878 (0.872, 0.884)	0.462 (0.441, 0.484)	0.599 (0.579, 0.620)	0.567 (0.548, 0.588)	0.052 (0.049, 0.055)
XGBoost	0.709 (0.700, 0.718)	0.776 (0.751, 0.800)	0.701 (0.691, 0.710)	0.242 (0.229, 0.256)	0.369 (0.351, 0.387)	0.312 (0.293, 0.331)	0.221 (0.215, 0.228)
KNN	0.715 (0.706, 0.723)	0.868 (0.847, 0.887)	0.696 (0.686, 0.705)	0.259 (0.246, 0.274)	0.399 (0.382, 0.417)	0.365 (0.349, 0.382)	0.077 (0.075, 0.079)
AdaBoost	0.629 (0.620, 0.639)	0.860 (0.839, 0.881)	0.601 (0.591, 0.611)	0.209 (0.198, 0.222)	0.337 (0.322, 0.353)	0.289 (0.274, 0.306)	0.096 (0.094, 0.099)

Data are presented as mean (95% CI). LR: Logistic Regression; XGBoost: eXtreme Gradient Boosting; KNN: K-nearest Neighbors; GBM: Gradient Boosting Machine; AdaBoost: Adaptive Boosting; SVM: Support Vector Machine; RF: Random Forest; MCC: Matthews correlation coefficient.

In the testing set, the RF model demonstrated excellent performance in discrimination and calibration, with an AUC of 0.946 and a Brier score of 0.052 (Table 1; Figures 4B,D). The RF model achieved the highest accuracy (0.875), specificity (0.878), precision (0.462), F1 score (0.599), and MCC (0.567; Table 1). The RF model achieved the lowest Log-Loss of 0.1847, indicating stable, superior performance compared to other models (Supplementary Table S4). The DeLong’s test results indicated that the RF model’s AUC significantly exceeded those of the other models (*p* < 0.001; Supplementary Table S6). The DCA results indicated that the RF model yielded a net benefit and exhibited superior performance compared with alternative models when the risk threshold was set between 3% and 98% (Figure 4F).

In summary, the RF model demonstrated superior performance compared to other models on both the training and testing sets, establishing it as the optimal choice. Additionally, in the internal temporal validation set, the RF model demonstrated strong generalizability with an AUC of 0.810 and a Brier score of 0.076, reflecting good discrimination and calibration (Supplementary Figure S3; Supplementary Table S7). Furthermore, to examine whether models built on balanced datasets outperform those built on imbalanced datasets, we repeated the model-building process on the balanced training dataset obtained after applying SMOTE and evaluated their performance on both the test set and the temporal validation set. The results indicated that the model performance did not improve significantly on the testing set (AUC = 0.879) and the temporal validation set (AUC = 0.744; Supplementary Figures S5, S6; Supplementary Table S8). Therefore, this study did not use the balanced data to construct the final model. Finally, we conducted a sensitivity analysis on the complete-case dataset before missing value imputation. The optimal model constructed was still the RF model (Supplementary Table S12), with AUCs of 1.000, 0.931, and 0.778 in the complete-case training set, testing set, and temporal validation set, respectively (Supplementary Figures S7–S9). These results were similar to those obtained using the imputed dataset for modeling.

### Model explanation

**Figure 5:**
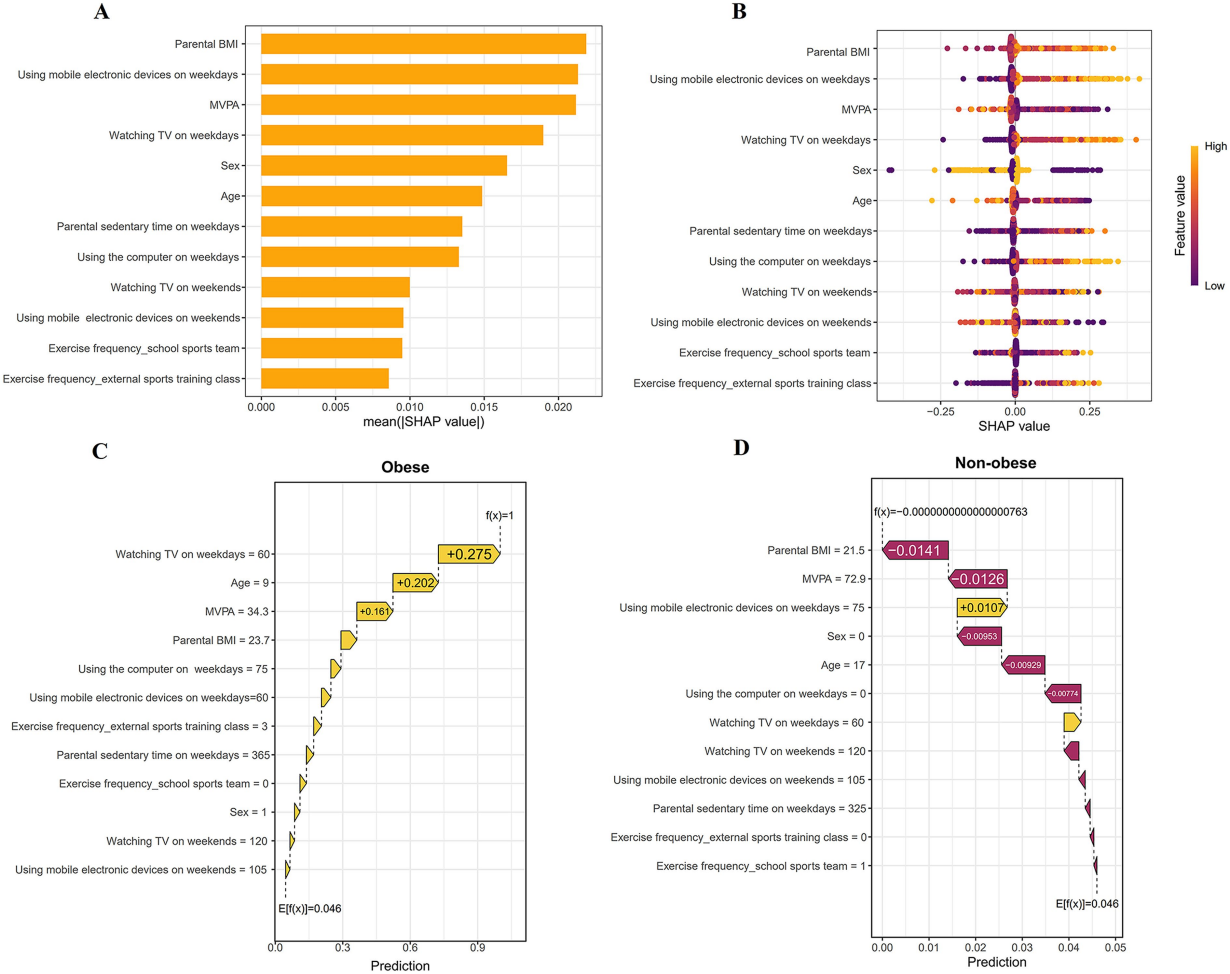
Global and local explanation of the model based on SHAP. **(A)** Summary bar plot. **(B)** Bee swarm plot. **(C,D)** Waterfall plot and risk evolution for each feature in individual children at varying obesity risk levels. BMI, body mass index; MVPA, moderate-to-vigorous physical activity.

The SHAP technique elucidates model outputs by quantifying the influence of each variable on predictions. It offers two distinct forms of explanations: a global feature-level explanation that shows overall feature importance, and a local individual-level explanation that focuses on the specific contributions to individual predictions. This combination offers a complete understanding of model performance from both general and specific perspectives. The global explanation reflects the overall performance of the model. As demonstrated in the bar chart in Figure 5A, the influence of each feature on the model was assessed through the mean SHAP values, which are presented in a descending sequence: Parental BMI, using mobile electronic devices on weekdays, MVPA, Watching TV on weekdays, Sex, Age, Parental sedentary time on weekdays, Using the computer on weekdays, Watching TV on weekends, Using mobile electronic devices on weekends, Exercise frequency—school sports team, Exercise frequency—external sports training class. The bee swarm plot illustrates the influence and direction of each feature on the model’s predictions (Figure 5B). For example, higher Parental BMI, increased use of mobile electronic devices on weekdays, and more watching TV on weekdays are associated with the model’s decision leaning toward obesity, while higher MVPA and greater age are associated with the model’s decision leaning toward non-obesity. Unlike global explanations, local explanations reflect the impact of each feature on the predicted outcomes for specific samples. The local explanation results for an individual predicted by the model to be obese are presented in Figure 5C. The features Watching TV on weekdays = 60, Age = 9, and MVPA = 34.3 contributed positively to the prediction result, with contributions of +0.275, +0.202, and +0.161, respectively. The local explanation results for an individual predicted by the model to be non-obese are presented in Figure 5D. The feature Using mobile electronic devices on weekends = 75 made a positive contribution of +0.0107 to the prediction result, while Parental BMI = 21.5 and MVPA = 72.9 made negative contributions of −0.0141 and −0.0126 to the prediction results, respectively.

### Implementation of the risk calculator application

**Figure 6:**
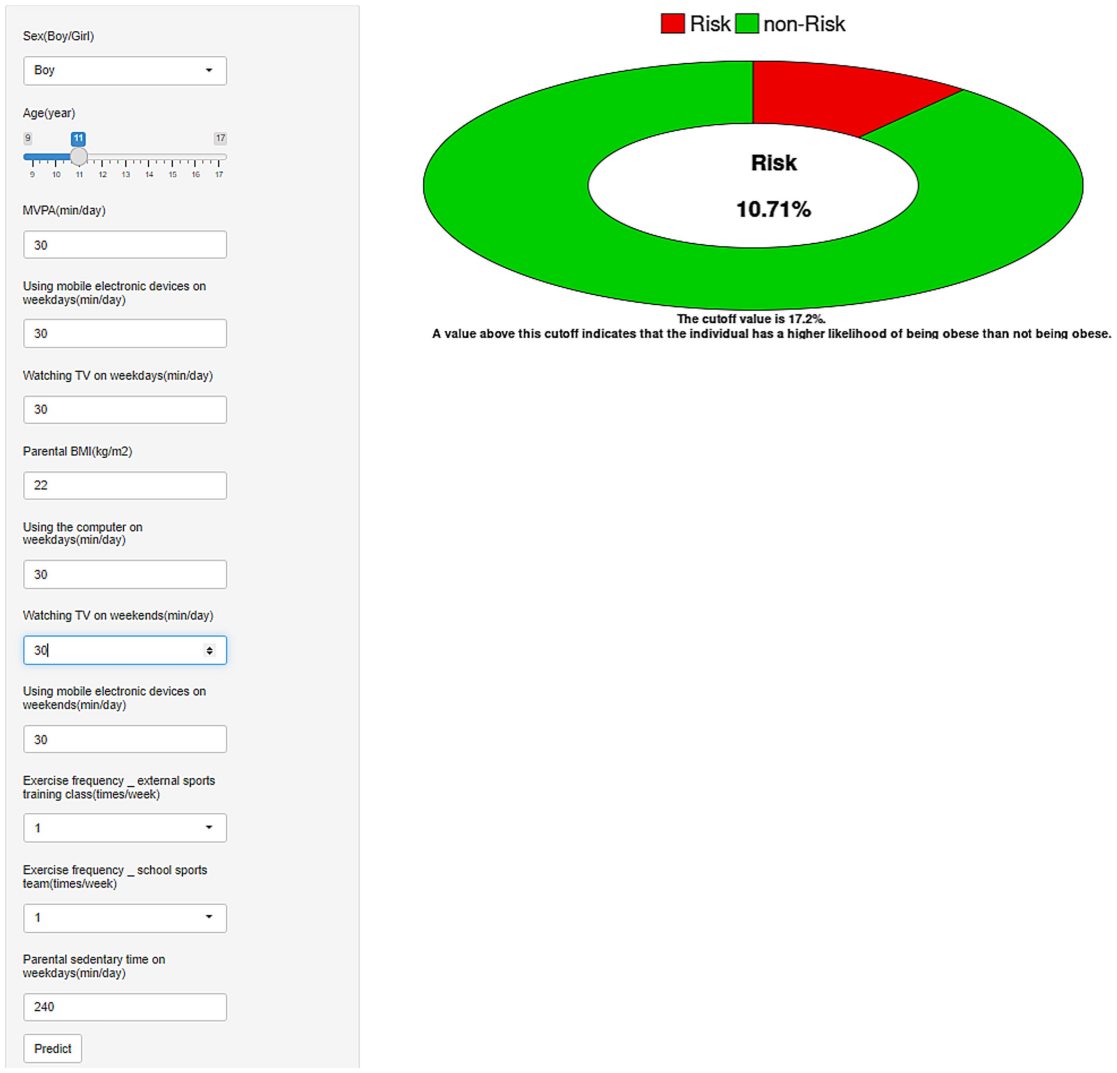
Obesity risk web-based calculator for children and adolescents. BMI, body mass index; MVPA, moderate-to-vigorous physical activity.

The RF model was incorporated into a web application to improve its practical usability (Figure 6). The risk calculator automatically computes the obesity risk score for children and adolescents by inputting the 12 required feature values into the predictive model on the webpage. The web risk calculator is available online at: https://predictivemodel123.shinyapps.io/make_web/.

## Discussion

As far as we know, this is the first study to construct and compare eight machine learning models to predict current obesity risk among children and adolescents in mainland China using nationally representative data. We identified a range of predictive risk factors, and using a large population sample of children and adolescents aged 9 to 17 from 31 administrative regions, developed obesity risk prediction models employing various machine learning algorithms. Our model provides an effective tool for parents, schools, and primary care centers to accurately assess obesity risk in children and adolescents, aiding obesity prevention in this demographic.

Currently, studies on predicting the risk of obesity in children and adolescents have been reported. However, the majority of studies sample from populations outside of China (8, 13, 14, 40, 41), which may misrepresent the traits of Chinese children and adolescents. Additionally, many of these studies use traditional logistic regression to construct models (9), which struggle to accommodate complex data and non-linear relationships, thereby affecting the accuracy of the models. Machine learning possesses the capability to identify intricate non-linear associations among variables (42) and is capable of handling large-scale complex datasets (43). Therefore, they are widely used to address prediction and classification problems (44, 45). By integrating nationally representative data from PAFCTYS, which include information on lifestyle behaviors and weight status, machine learning algorithms can be used to develop accurate obesity risk prediction models. This study employed eight machine learning algorithms to develop a model predicting obesity risk. The RF model achieved the highest AUC among all evaluated models, demonstrating superior discrimination and calibration in both the training and testing sets. Similar to this study, previous research has demonstrated the excellent performance of the RF model in predicting disease risk (46–48). Random forest consists of multiple individual decision trees that exhibit significant diversity, thereby mitigating overfitting (49). Additionally, the RF model performs well at handling high-dimensional data and demonstrates strong robustness to outliers and noise (50). The study’s final RF model incorporated 12 readily obtainable features, offering a promising tool for large-scale obesity risk screening in children and adolescents without requiring complex measurements.

The process of feature selection plays an essential role in developing predictive models, significantly influencing both the model’s effectiveness and its potential applications (51, 52). Presently, no formal guidelines or standards exist for feature selection in risk prediction model development. An inadequate number of features can impair a model’s predictive accuracy, while an excessive number can lead to complex models susceptible to overfitting, hindering generalization and practical implementation. Therefore, we used LASSO combined with RFE for feature selection, ultimately including 12 feature variables to develop the predictive model. Similar to previous studies (52, 53), the predictive model constructed after feature selection strikes a good balance between predictive performance and simplicity, making it easy to apply in real-world settings.

We employed multiple metrics to compare the discrimination, calibration, and clinical utility of different machine learning models. The AUC, accuracy, sensitivity, and specificity are classic indicators for evaluating the discriminative ability of prediction models. The RF model achieved the highest AUC, accuracy, and sensitivity in both training and testing datasets. Despite the SVM model exhibiting marginally higher specificity in the testing set, the RF model overall demonstrates superior discriminative power. Additionally, we utilized the F1 score and MCC to evaluate the models’ discriminative performance. MCC is considered a more effective metric than F1 score and accuracy for assessing performance on imbalanced datasets (54). The RF model demonstrated superior discriminative performance, achieving the highest F1 score and MCC in both training and testing datasets. Although the AUC in this study decreased from 0.946 on the testing set to 0.810 on the temporal validation set, this is, to some extent, explainable and acceptable. Compared to the testing set, the decline in AUC for the temporal validation set is not uncommon in previous studies (55, 56) and is mainly determined by the nature of the temporal validation set. One possible reason for the AUC decline in the temporal validation set in this study is changes in the social environment, including overall BMI level increases due to improved economic conditions and rising smartphone penetration rates (57, 58), which may affect the degree of association between feature variables and outcomes in the temporal validation set.

Second, COVID-19 pandemic-related factors may also affect the AUC of the temporal validation set, as the temporal validation set used data from 2020. Specifically, children and adolescents’ behavioral patterns may have been inevitably affected during COVID-19, such as decreased physical activity and prolonged sedentary behavior (59–61). This narrowed the differences in physical activity and sedentary behavior between previously active and inactive adolescents, thereby reducing the prediction model’s ability to distinguish obesity based on these behavioral indicators. Additionally, different provinces and regions of China were affected by COVID-19 to varying degrees, leading to inconsistent lockdown durations. This, to some extent, increased the heterogeneity in the associations between physical activity, sedentary behavior, and obesity among children and adolescents, thereby increasing the classification difficulty for the prediction model and leading to a certain degree of AUC decline in the temporal validation set. However, even the AUC in the validation set declined compared to the testing set, when using data from the 2020 COVID-19 pandemic period as the temporal validation set, the model’s discriminative ability still remained at a good level (AUC > 0.8), which to some extent demonstrates that the model has considerable temporal stability.

Calibration reflects the consistency between the true probability of outcomes and the probabilities predicted by the model, representing another important dimension for evaluating prediction model performance. However, current research on predicting overweight and obesity in children and adolescents often prioritizes discriminative performance over calibration. In the present investigation, we employed the Brier Score and Log Loss metrics to assess model calibration. By combining the performance of these two metrics in both the testing and training datasets, we confirmed that the final RF model not only demonstrates excellent discriminative ability but also exhibits good calibration. Furthermore, we performed a DCA to evaluate the model’s practical applicability. The findings demonstrate that the RF model provides a superior net benefit for obesity risk assessment compared to other models when the risk threshold is between 3 and 98%.

However, it should be noted that, despite relatively high AUC, sensitivity, and specificity, the precision in this study was relatively low in the testing set (Precision = 0.462), suggesting that appropriate caution is needed when interpreting the diagnostic results of the prediction model. Nevertheless, this does not diminish the potential of the prediction model developed in this study as a screening tool for childhood and adolescent obesity. First, low precision when constructing prediction models for diseases with low prevalence is a common problem currently faced in prediction model development (62, 63). In the real world, the prevalence of obesity is relatively low. Taking China as an example, only approximately 10% of children and adolescents are obese. Due to the large number of negative samples, even a small number of false positives can significantly reduce precision, and similar studies have commonly reported low precision. For example, the overweight and obesity prediction model constructed by Ziauddeen et al. achieved a precision of 55.0% at a 30% risk threshold (64). In another prediction model study of obesity in the 12–24 age group, the precision ranged from 8.5 to 40.7% across different probability thresholds (65). Second, the prediction model developed in this study is not intended to serve as a diagnostic tool for obesity, but rather as a screening tool to identify potential high-risk populations for obesity in large-scale screening programs, preparing for subsequent tiered management or interventions. Finally, unlike false positives in predictions for diseases such as cancer and heart disease, false positives in obesity prediction are less likely to cause excessive panic and essentially do not involve concerns about diagnostic costs. Instead, they can, to some extent, enhance obesity risk prevention awareness among screened individuals, prompting them to pay closer attention to their weight status and reflect on whether they have unhealthy behaviors. Therefore, given that the model in this study has high AUC, sensitivity, and specificity, the misclassification due to low precision is largely acceptable in practical applications for the initial screening of obesity-risk populations.

With advances and iterations in technology, the ability of machine learning models to make accurate predictions continues to improve. However, the complexity of model structures has increased, leading to the phenomenon known as the ‘black box’ characteristic (66). This lack of transparency prevents users from understanding the underlying reasons and the logical steps that lead to the model’s decisions (67), which significantly undermines clinicians’ or users’ trust in the model and hinders its widespread application in practice. This study employed the SHAP method to address challenges associated with black box models. SHAP provides feature importance explanations both globally across the dataset and locally for individual samples (68, 69). SHAP can demonstrate the average influence of particular features on the model’s output and assess the direction and strength of feature impacts on individual predictions. The SHAP results indicate that the top five features with the greatest influence on the model’s output in this study are, in order: parental BMI, MVPA, using mobile electronic devices on weekdays, watching TV on weekdays, and sex. The summary plot also indicates that higher parental BMI, the use of mobile electronic devices on weekdays, and watching TV on weekdays are associated with an increased likelihood of obesity, whereas elevated levels of MVPA are associated with a reduced risk of obesity. This is consistent with previous epidemiological research findings (70, 71). Additionally, to enhance the usability of the prediction model, we deployed an online app based on the Shinyapps platform, allowing users to conveniently access the model for obesity risk prediction anytime and anywhere.

Although this study has the aforementioned advantages, it is crucial to recognize its limitations as well. Firstly, we did not include all possible variables related to obesity, which may result in a certain degree of loss in predictive performance. However, compared to previous studies that included overly complex or difficult-to-obtain feature variables (11, 72), this study constructed a predictive model using a simpler and relatively smaller number of features. This way not only preserved strong model accuracy but also improved the model’s ability to generalize effectively. Secondly, the model was constructed using data from Chinese children and adolescents. Despite having undergone temporal validation, its generalizability to other populations may remain uncertain. Thirdly, while this study includes school-aged children and adolescents aged 9 to 17, who comprise the majority of this population, the PAFCTYS does not include lower-grade elementary students (grades 1–3). Future research should consider expanding the age range. The significant variability in reading comprehension skills among lower-grade elementary students makes questionnaire surveys challenging, which is why previous studies often focus on middle school or upper-grade elementary students (11, 73). Despite the aforementioned limitations, the model’s excellent performance remains evident and suggests significant potential for broad applicability.

In conclusion, we successfully developed an obesity risk prediction model for Chinese children and adolescents aged 9 to 17 using interpretable machine learning based on readily obtainable feature variables from the PAFCTYS. The final RF model demonstrated strong performance across the training, testing, and temporal validation sets. Integrating this predictive model into a web application enables health organizations, school health staff, and parents to quickly and accurately evaluate current obesity risk in children and adolescents, laying the groundwork for targeted interventions and health education initiatives.

## Data Availability

The original contributions presented in the study are included in the article/Supplementary material, further inquiries can be directed to the corresponding authors.
