# Supplementary material for: Development and validation of an explainable machine learning-based risk prediction model for obesity in Chinese children and adolescents: a population-based study
Source: Front Nutr. 2026 May 5;13:1816724. doi: 10.3389/fnut.2026.1816724 (PMC13183532; doi:10.3389/fnut.2026.1816724)
Supplement: Supplementary file 1 [file Supplementary_file_1.docx]

**Supplementary Material**

**Table S1** Variables Included in the Study. P2

**Table S2** Basic Characteristics of Participants. P3-P4

**Table S3** Hyperparameters for eight models. P4

**Table S4** Log-Loss of different machine learning models in training and testing sets. P5

**Table S5** Delong test results of AUC differences between various machine learning models in the training set. P5

**Table S6** Delong test results of AUC differences between various machine learning models in the testing set. P5

**Table S7** Performance metrics of various machine learning models in the internal validation set. P6

**Table S8** Log-Loss of different machine learning models in training and testing sets after SMOTE. P6

**Table S9** Imputed variables in the training set and their respective missing rates. P6-P7

**Table S10** Imputed variables in the testing set and their respective missing rates. P8

**Table S11** Imputed variables in the internal validation set and their respective missing rates. P8

**Table S12** Performance metrics of various machine learning models in the complete-case training set and testing set. P9-P10

**Figure S1** Confusion matrix of the RF model in the training set. P11

**Figure S2** Confusion matrix of the RF model in the testing set. P12

**Figure S3** ROC curves of various machine learning models in the internal validation set. P13

**Figure S4** Outcome distribution before and after SMOTE. P13

**Figure S5** ROC curves of various machine learning models trained after SMOTE in the testing set. P14

**Figure S6** ROC curves of various machine learning models trained after SMOTE in the temporal validation set. P14

**Figure S7** ROC curves of various machine learning models in the complete-case training set. P15

**Figure S8** ROC curves of various machine learning models in the complete-case testing set. P15

**Figure S9** ROC curves of various machine learning models in the complete-case temporal validation set. P16

**Supplementary methods** P17-P19

**Table S1 Variables Included in the Study.**

|  | **Variable** |
| --- | --- |
| Student |  |
|  | Sex |
|  | Age |
|  | Exercise intention |
|  | Watching TV on weekdays |
|  | Using mobile electronic devices on weekdays |
|  | Using the computer on weekdays |
|  | Doing homework on weekdays |
|  | Watching TV on weekends |
|  | Using mobile electronic devices on weekends |
|  | Using the computer on weekends |
|  | Doing homework on weekends |
|  | MVPA frequency on weekday |
|  | MVPA frequency on weekend |
|  | Muscle-strengthening exercise frequency |
|  | MPA |
|  | VPA |
|  | MVPA |
|  | Walking |
|  | Sleep duration |
|  | Exercise frequency _ physical education class |
|  | Exercise frequency _ morning exercise |
|  | Exercise frequency _ recess activities |
|  | Exercise frequency _ school sports team |
|  | Exercise frequency _ school sports club |
|  | Exercise frequency _ extracurricular sports competitions |
|  | Exercise frequency _ external sports training class |
|  | Exercise frequency _ self-exercise |
| Parent |  |
|  | Parental attitude toward exercise |
|  | Parental MVPA frequency on weekdays |
|  | Parental MVPA frequency on weekends |
|  | Parental muscle-strengthening exercise frequency |
|  | Parental sedentary time on weekdays |
|  | Parental sedentary time on weekends |
|  | Parental MPA |
|  | Parental VPA |
|  | Parental MVPA |
|  | Parental walking |
|  | Parental BMI |

Abbreviations: BMI: body mass index; MVPA: moderate to-vigorous physical activity; VPA: vigorous physical activity; MPA: moderate physical activity.

**Table S2 Basic Characteristics of Participants.**

| **Variable** | **Non-obese(n=31182)** | **Obese(n=3834)** | **P** |
| --- | --- | --- | --- |
| **Student** |  |  |  |
| Sex |  |  | <0.001 |
| Boys n(%) | 16042(51.4) | 2504(65.3) |  |
| Girls n(%) | 15140(48.6) | 1330(34.7) |  |
| Age (years) | 13.0(11.0,14.0) | 11.0(10.0,13.0) | <0.001 |
| Exercise intention | 2(1,3) | 2(1,3) | <0.001 |
| Watching TV on weekdays (min/day) | 0.0(0.0,30.0) | 30.0(0.0,60.0) | <0.001 |
| Using mobile electronic devices on weekdays (min/day) | 30.0(0.0,30.0) | 30.0(30.0,60.0) | <0.001 |
| Using the computer on weekdays (min/day) | 0.0(0.0,30.0) | 30.0(30.0,60.0) | <0.001 |
| Doing homework on weekdays (min/day) | 120.0(60.0,120.0) | 120.0(60.0,120.0) | 0.725 |
| Watching TV on weekends (min/day) | 30.0(0.0,60.0) | 60.0(30.0,60.0) | <0.001 |
| Using mobile electronic devices on weekends (min/day) | 30.0(30.0,60.0) | 60.0(30.0,105.0) | <0.001 |
| Using the computer on weekends (min/day) | 30.0(0.0,60.0) | 30.0(0.0,60.0) | <0.001 |
| Doing homework on weekends (min/day) | 120.0(60.0,180.0) | 120.0(60.0,180.0) | 0.380 |
| MVPA frequency on weekday | 3(2,4) | 2(1,4) | <0.001 |
| MVPA frequency on weekend | 1(1,2) | 1(1,2) | 0.017 |
| Muscle-strengthening exercise frequency | 3(1,4) | 2(1,4) | <0.001 |
| MPA (min/day) | 25.7(11.4,50.0) | 20.0(8.5,34.2) | <0.001 |
| VPA (min/day) | 25.7(10.7,46.4) | 17.1(9.2,34.2) | <0.001 |
| MVPA (min/day) | 54.2(27.1,94.2) | 40.0(22.8,67.8) | <0.001 |
| Walking (min/day) | 30.0(15.0,64.2) | 28.5(11.4,60.0) | <0.001 |
| Sleep duration (h/day) | 7.5(7.5,8.5) | 8.5(7.5,8.5) | <0.001 |
| Exercise Frequency _ Physical Education Classes | 3(2,3) | 3(2,3) | 0.555 |
| Exercise Frequency _ Morning Exercise | 5(2,5) | 5(1,5) | <0.001 |
| Exercise Frequency _ Recess Activities | 5(2,5) | 5(2,5) | 0.405 |
| Exercise Frequency _ School Sports Team | 0(0,1) | 0(0,1) | <0.001 |
| Exercise Frequency _ School Sports Club | 0(0,1) | 0(0,1) | <0.001 |
| Exercise Frequency _ Extracurricular Sports Competitions | 0(0,2) | 0(0,2) | <0.001 |
| Exercise Frequency _ External Sports Training Classes | 0(0,1) | 0(0,1) | <0.001 |
| Exercise Frequency _ Self-Exercise | 3(2,5) | 3(2,5) | <0.001 |
| **Parent** |  |  |  |
| Parental attitude toward exercise | 3(2,3) | 3(2,3) | 0.648 |
| Parental MVPA frequency on weekdays (min/day) | 2(1,3) | 2(1,3) | 0.058 |
| Parental MVPA frequency on weekends (min/day) | 1(0,1) | 1(0,1) | 0.002 |
| Parental muscle-strengthening exercise frequency | 0(0,2) | 0(0,2) | <0.001 |
| Parental sedentary time on weekday (min/day) | 90.0(0.0,240.0) | 120(0.0,255.0) | <0.001 |
| Parental sedentary time on weekend (min/day) | 90.0(0.0,200.0) | 90.0(0.0,210.0) | 0.009 |
| Parental MPA (min/day) | 8.5(0.0,30.0) | 8.5(0.0,25.7) | <0.001 |
| Parental VPA (min/day) | 8.5(0.0,25.7) | 8.5(0.0,25.7) | 0.038 |
| Parental MVPA (min/day) | 22.8(0.0,60.0) | 21.4(0.0,57.1) | 0.003 |
| Parental Walking (min/day) | 32.1(10.0,74.2) | 30.0(10.0,70.0) | 0.239 |
| Parental BMI (kg/m^2^) | 22.1(20.1,24.4) | 23.8(21.6,26.3) | <0.001 |

Note: Data are presented as medians with interquartile ranges (IQR) and compared using the Mann–Whitney U test or the Kruskal–Wallis H test

Abbreviations: BMI: body mass index; MVPA: moderate to-vigorous physical activity; VPA: vigorous physical activity; MPA: moderate physical activity.

**Table S3 Hyperparameters for eight models.**

| **Model** | **Hyperparameter** |
| --- | --- |
| Logistic Regression (LR) | none |
| Random Forest (RF) | mtry = 2, ntree=300 |
| Support Vector Machine (SVM) | sigma =0.1, C = 0.5 |
| K-Nearest Neighbors (KNN) | k = 15, distance=1, kernel = "optimal" |
| Gradient boosting machines (GBM) | n.trees = 100, interaction.depth = 3, shrinkage = 0.1, n.minobsinnode = 5 |
| eXtreme Gradient Boosting (XGBoost) | eta = 0.1, max_depth = 5, gamma =0.5, colsample_bytree= 0.5, min_child_weight= 1, subsample=0.6, nrounds=10 |
| Adaptive Boosting (AdaBoost ) | mfinal = 2, maxdepth = 5, coeflearn = "Zhu" |
| Neural Network (NNET) | size = 3, decay = 0.6 |

**Table S4 Log-Loss of different machine learning models in training and testing sets.**

| **Model** | **Log_Loss_Train** | **Log_Loss_Test** |
| --- | --- | --- |
| LR | 0.2837 | 0.3217 |
| KNN | 0.2003 | 0.3059 |
| AdaBoost | 0.7152 | 0.7757 |
| NNET | 0.2913 | 0.2958 |
| RF | 0.0939 | 0.1847 |
| GBM | 0.2616 | 0.2720 |
| SVM | 0.2858 | 0.3110 |
| XGBoost | 0.6345 | 0.6355 |

Abbreviations: LR: Logistic Regression; SVM: Support Vector Machine; RF: Random Forest; XGBoost: eXtreme Gradient Boosting; KNN: K-nearest Neighbors; GBM: Gradient Boosting Machine; NNET: Neural Network; AdaBoost: Adaptive Boosting.

**Table S5 Delong test results of AUC differences between various machine learning models in the training set.**

| **New model-**  **Baseline model** | **New model** | **Baseline model** | **Difference of AUC** | **95% CI** | **Z statistic** | **P** |
| --- | --- | --- | --- | --- | --- | --- |
| RF-LR | 1 | 0.801 | 0.199 | 0.191 to 0.208 | 46.203 | <0.0001 |
| RF-SVM | 1 | 0.829 | 0.171 | 0.162 to 0.181 | 35.510 | <0.0001 |
| RF-KNN | 1 | 0.945 | 0.0546 | 0.051 to 0.057 | 36.418 | <0.0001 |
| RF-AdaBoost | 1 | 0.737 | 0.263 | 0.256 to 0.270 | 73.568 | <0.0001 |
| RF- XGBoost | 1 | 0.829 | 0.171 | 0.164 to 0.179 | 43.314 | <0.0001 |
| RF-GBM | 1 | 0.846 | 0.154 | 0.147 to 0.161 | 42.808 | <0.0001 |
| RF-NNET | 1 | 0.794 | 0.206 | 0.197 to 0.214 | 47.382 | <0.0001 |

Abbreviations: LR: Logistic Regression; SVM: Support Vector Machine; RF: Random Forest; XGBoost: eXtreme Gradient Boosting ; KNN: K-nearest Neighbors; GBM: Gradient Boosting Machine; NNET: Neural Network; AdaBoost: Adaptive Boosting.

**Table S6 Delong test results of AUC differences between various machine learning models in the testing set.**

| **New model-**  **Baseline model** | **New model** | **Baseline model** | **Difference of AUC** | **95% CI** | **Z statistic** | **P** |
| --- | --- | --- | --- | --- | --- | --- |
| RF-LR | 0.946 | 0.756 | 0.190 | 0.177 to 0.202 | 28.905 | <0.0001 |
| RF-SVM | 0.946 | 0.705 | 0.241 | 0.162 to 0.181 | 28.527 | <0.0001 |
| RF-KNN | 0.946 | 0.852 | 0.0944 | 0.086 to 0.102 | 23.238 | <0.0001 |
| RF-AdaBoost | 0.946 | 0.731 | 0.215 | 0.205 to 0.226 | 39.874 | <0.0001 |
| RF- XGBoost | 0.946 | 0.811 | 0.135 | 0.124 to 0.146 | 24.803 | <0.0001 |
| RF-GBM | 0.946 | 0.829 | 0.117 | 0.107 to 0.126 | 24.128 | <0.0001 |
| RF-NNET | 0.946 | 0.783 | 0.163 | 0.151 to 0.175 | 26.454 | <0.0001 |

Abbreviations: LR: Logistic Regression; SVM: Support Vector Machine; RF: Random Forest; XGBoost: eXtreme Gradient Boosting ; KNN: K-nearest Neighbors; GBM: Gradient Boosting Machine; NNET: Neural Network; AdaBoost: Adaptive Boosting.

**Table S7 Performance metrics of various machine learning models in the internal validation set.**

| **Model** | **Accuracy** | **Sensitivity** | **Specificity** | **Precision** | **F1** | **MCC** | **Brier score** |
| --- | --- | --- | --- | --- | --- | --- | --- |
| LR | 0.591 | 0.784 | 0.568 | 0.175 | 0.286 | 0.216 | 0.115024 |
| SVM | 0.793 | 0.421 | 0.837 | 0.232 | 0.299 | 0.201 | 0.090064 |
| GBM | 0.732 | 0.713 | 0.734 | 0.239 | 0.358 | 0.295 | 0.085867 |
| NNET | 0.669 | 0.787 | 0.655 | 0.211 | 0.332 | 0.277 | 0.087665 |
| RF | 0.810 | 0.762 | 0.704 | 0.232 | 0.355 | 0.301 | 0.076174 |
| XGBoost | 0.629 | 0.844 | 0.603 | 0.199 | 0.323 | 0.276 | 0.220698 |
| KNN | 0.696 | 0.713 | 0.694 | 0.214 | 0.329 | 0.261 | 0.087332 |
| AdaBoost | 0.670 | 0.784 | 0.656 | 0.211 | 0.332 | 0.276 | 0.093014 |

Abbreviations: LR: Logistic Regression; SVM: Support Vector Machine; RF: Random Forest; XGBoost: eXtreme Gradient Boosting ; KNN: K-nearest Neighbors; GBM: Gradient Boosting Machine; NNET: Neural Network; AdaBoost: Adaptive Boosting; MCC: Matthews correlation coefficient.

**Table S8 Log-Loss of different machine learning models in training and testing sets after SMOTE.**

| **Model** | **Log_Loss_Train** | **Log_Loss_Test** |
| --- | --- | --- |
| LR | 0.5413 | 0.5612 |
| KNN | 0.000 | 8.5275 |
| AdaBoost | 3.2415 | 3.7249 |
| NNET | 0.5226 | 0.5519 |
| RF | 0.1575 | 0.4432 |
| GBM | 0.4389 | 0.4718 |
| SVM | 0.4494 | 0.5061 |
| XGBoost | 0.6722 | 0.6746 |

Abbreviations: LR: Logistic Regression; SVM: Support Vector Machine; RF: Random Forest; XGBoost: eXtreme Gradient Boosting ; KNN: K-nearest Neighbors; GBM: Gradient Boosting Machine; NNET: Neural Network; AdaBoost: Adaptive Boosting.

**Table S9 Imputed variables in the training set and their respective missing rates.**

|  | **Variable** | **Missing rate** |
| --- | --- | --- |
| Student |  |  |
|  | Exercise intention | 0.22% |
|  | Watching TV on weekdays | 0.17% |
|  | Using mobile electronic devices on weekdays | 0.18% |
|  | Using the computer on weekdays | 0.22% |
|  | Doing homework on weekdays | 0.16% |
|  | Watching TV on weekends | 0.22% |
|  | Using mobile electronic devices on weekends | 0.21% |
|  | Using the computer on weekends | 0.29% |
|  | Doing homework on weekends | 0.21% |
|  | MVPA frequency on weekday | 0.19% |
|  | MVPA frequency on weekend | 0.32% |
|  | Muscle-strengthening exercise frequency | 0.26% |
|  | Walking | 0.05% |
|  | Sleep duration | 0.31% |
|  | Exercise frequency _ physical education class | 0.73% |
|  | Exercise frequency _ morning exercise | 0.66% |
|  | Exercise frequency _ recess activities | 0.80% |
|  | Exercise frequency _ school sports team | 1.67% |
|  | Exercise frequency _ school sports club | 1.68% |
|  | Exercise frequency _ extracurricular sports competitions | 1.56% |
|  | Exercise frequency _ external sports training class | 1.64% |
|  | Exercise frequency _ self-exercise | 0.46% |
| Parent |  |  |
|  | Parental attitude toward exercise | 0.81% |
|  | Parental MVPA frequency on weekdays | 0.72% |
|  | Parental MVPA frequency on weekends | 0.61% |
|  | Parental muscle-strengthening exercise frequency | 0.69% |
|  | Parental sedentary time on weekdays | 9.38% |
|  | Parental sedentary time on weekends | 9.38% |
|  | Parental MPA | 9.46% |
|  | Parental VPA | 6.60% |
|  | Parental MVPA | 11.2% |
|  | Parental walking | 13.3% |

**Table S10 Imputed variables in the testing set and their respective missing rates.**

|  | **Variable** | **Missing rate** |
| --- | --- | --- |
| Student |  |  |
|  | Watching TV on weekdays | 0.19% |
|  | Using mobile electronic devices on weekdays | 0.22% |
|  | Using the computer on weekdays | 0.27% |
|  | Watching TV on weekends | 0.17% |
|  | Using mobile electronic devices on weekends | 0.17% |
|  | Exercise frequency _ school sports team | 1.76% |
|  | Exercise frequency _ external sports training class | 1.58% |
| Parent |  |  |
|  | Parental sedentary time on weekdays | 9.44% |

**Table S11 Imputed variables in the internal validation set and their respective missing rates.**

|  | **Variable** | **Missing rate** |
| --- | --- | --- |
| Student |  |  |
|  | Watching TV on weekdays | 0.40% |
|  | Using mobile electronic devices on weekdays | 0.46% |
|  | Using the computer on weekdays | 0.56% |
|  | Watching TV on weekends | 0.54% |
|  | Using mobile electronic devices on weekends | 0.62% |
|  | Exercise frequency _ school sports team | 2.87% |
|  | Exercise frequency _ external sports training class | 2.63% |
| Parent |  |  |
|  | Parental sedentary time on weekdays | 17.7% |

**Table S12 Performance metrics of various machine learning models in the complete-case training set and testing set.**

| **Model** | **Accuracy** | **Sensitivity** | **Specificity** | **Precision** | **F1 score** | **MCC** | **Brier score** |
| --- | --- | --- | --- | --- | --- | --- | --- |
| **Training set** |  |  |  |  |  |  |  |
| LR | 0.75 (0.744, 0.756) | 0.723 (0.703, 0.742) | 0.754 (0.747, 0.760) | 0.268 (0.255, 0.279) | 0.391 (0.375, 0.405) | 0.326(0.311, 0.342) | 0.082 (0.080, 0.084) |
| SVM | 0.888 (0.882, 0.892) | 0.714 (0.694, 0.734) | 0.909 (0.905, 0.913) | 0.496 (0.476, 0.513) | 0.585 (0.568, 0.600) | 0.534 (0.516, 0.551) | 0.079 (0.076, 0.082) |
| GBM | 0.757 (0.750, 0.763) | 0.801 (0.782, 0.817) | 0.752 (0.745, 0.758) | 0.287 (0.275, 0.298) | 0.423 (0.408, 0.436) | 0.375 (0.360, 0.389) | 0.076 (0.074,0.078) |
| Neural Network | 0.721 (0.714, 0.727) | 0.808 (0.790, 0.825) | 0.710 (0.703, 0.716) | 0.258 (0.246, 0.269) | 0.391 (0.377, 0.404) | 0.341 (0.327, 0.355) | 0.081 (0.079, 0.082) |
| RF | 0.997 (0.996, 0.998) | 0.997 (0.994, 0.999) | 0.998 (0.997, 0.999) | 0.982 (0.976, 0.986) | 0.989 (0.986, 0.992) | 0.988 (0.984, 0.991) | 0.022 (0.021, 0.023) |
| XGBoost | 0.753 (0.747, 0.759 | 0.754 (0.734, 0.774) | 0.753 (0.747, 0.760) | 0.276 (0.263, 0.288) | 0.404 (0.389, 0.418) | 0.347 (0.331, 0.362) | 0.221 (0.219, 0.222) |
| KNN | 0.828 (0.823, 0.833) | 1.000 (1.000, 1.000) | 0.807 (0.801, 0.813) | 0.392 (0.379, 0.406) | 0.563 (0.550, 0.577) | 0.563 (0.551, 0.573) | 0.061 (0.059, 0.063) |
| AdaBoost | 0.718 (0.712, 0.725) | 0.792 (0.775, 0.810) | 0.709 (0.702, 0.716) | 0.253 (0.242, 0.264) | 0.384 (0.370, 0.397) | 0.330 (0.317, 0.345) | 0.092 (0.090, 0.094) |
| **Testing set** |  |  |  |  |  |  |  |
| LR | 0.692 (0.682, 0.701) | 0.766 (0.740, 0.792) | 0.683 (0.672, 0.692) | 0.227 (0.213, 0.242) | 0.350 (0.332, 0.369) | 0.289 (0.271, 0.310) | 0.085 (0.082, 0.088) |
| SVM | 0.780 (0.771, 0.788) | 0.668 (0.638, 0.697) | 0.793 (0.784, 0.802) | 0.281 (0.264, 0.299) | 0.396 (0.376, 0.417) | 0.328 (0.307, 0.351) | 0.083 (0.080, 0.086) |
| GBM | 0.750 (0.741, 0.759) | 0.774 (0.749, 0.801) | 0.747 (0.738, 0.756) | 0.271 (0.255, 0.287) | 0.401 (0.383, 0.422) | 0.351 (0.331, 0.371) | 0.078 (0.075, 0.081) |
| Neural Network | 0.711 (0.701, 0.720) | 0.800 (0.775, 0.825) | 0.700 (0.690, 0.709) | 0.244 (0.231, 0.260) | 0.374 (0.357, 0.394 | 0.325 (0.306, 0.344) | 0.083 (0.080, 0.086) |
| RF | 0.872 (0.865, 0.878 | 0.831 (0.808, 0.855) | 0.877 (0.870, 0.884) | 0.450 (0.427, 0.474) | 0.583 (0.562, 0.606) | 0.550 (0.527, 0.571) | 0.059 (0.056, 0.062) |
| XGBoost | 0.735 (0.726, 0.744) | 0.733 (0.707, 0.761) | 0.735 (0.726, 0.744) | 0.251 (0.237, 0.267) | 0.374 (0.356, 0.394) | 0.313 (0.293, 0.334) | 0.221 (0.213, 0.228) |
| KNN | 0.733 (0.724, 0.742) | 0.804 (0.780, 0.828) | 0.724 (0.715, 0.733) | 0.261 (0.247, 0.278) | 0.394 (0.376, 0.415) | 0.348 (0.329, 0.367) | 0.079 (0.076, 0.082) |
| AdaBoost | 0.704 (0.694, 0.713) | 0.765 (0.739, 0.792) | 0.696 (0.686, 0.706) | 0.234 (0.221, 0.250) | 0.358 (0.341, 0.379) | 0.300 (0.281, 0.320) | 0.093 (0.090, 0.096) |

Note: Data are presented as mean (95% CI).

Abbreviations: LR: Logistic Regression; XGBoost: eXtreme Gradient Boosting ; KNN: K-nearest Neighbors; GBM: Gradient Boosting Machine; AdaBoost: Adaptive Boosting; SVM: Support Vector Machine; RF: Random Forest; MCC: Matthews correlation coefficient.


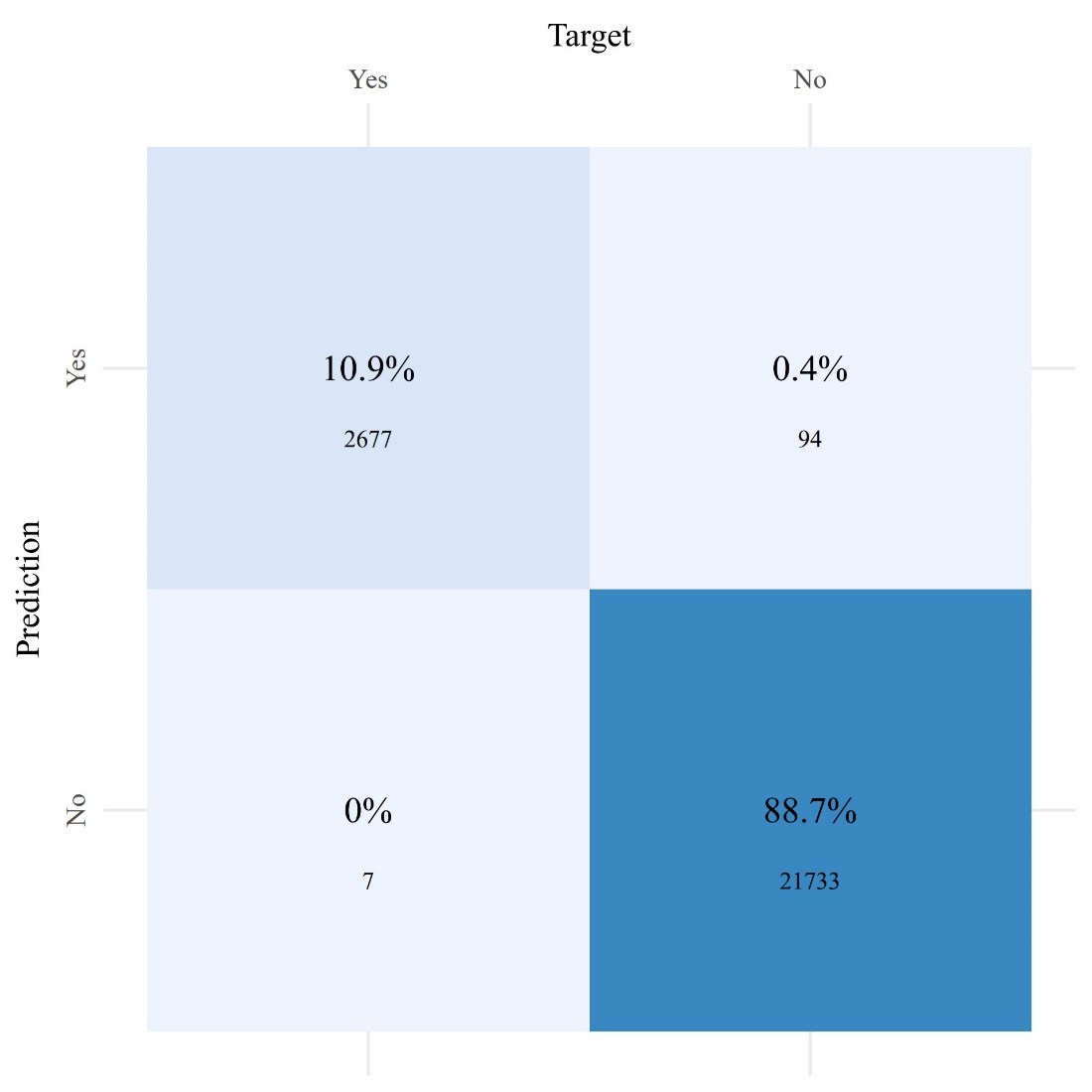


**Figure S1 Confusion matrix of the RF model in the training set.**


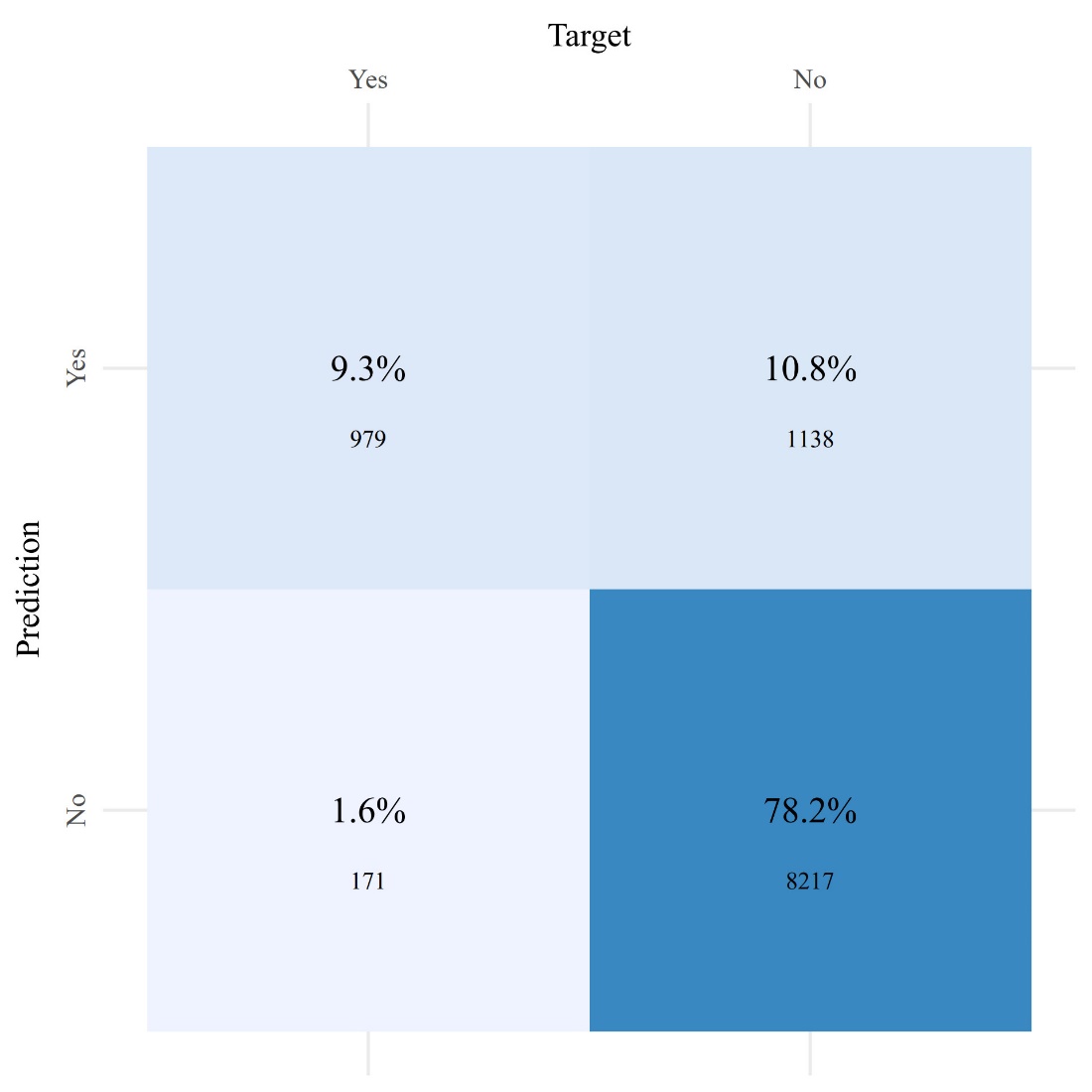


**Figure S2 Confusion matrix of the RF model in the testing set.**


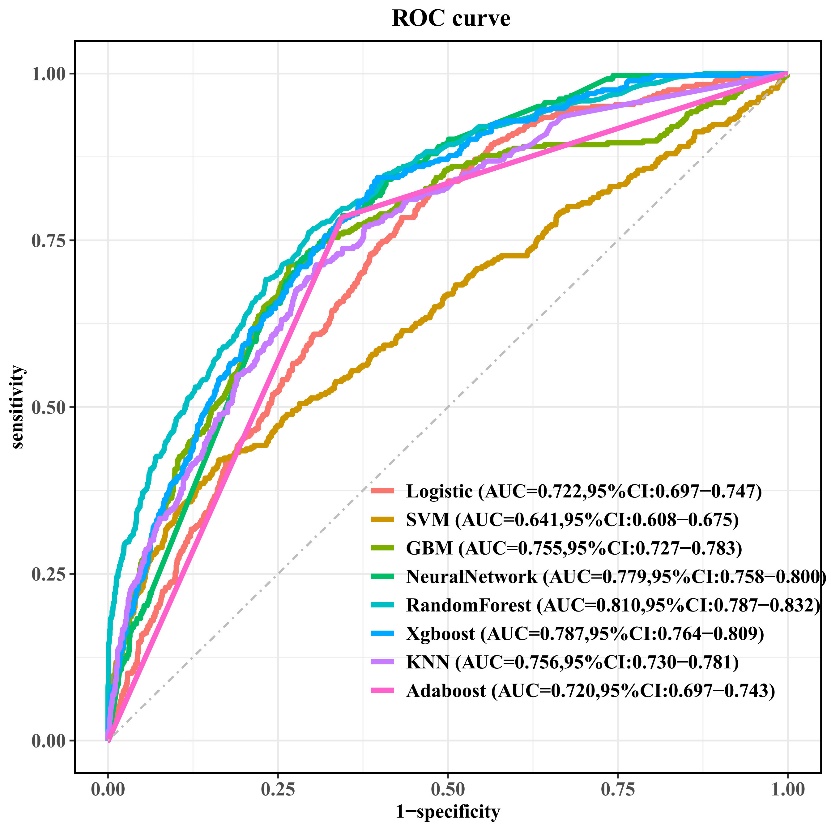


**Figure S3 ROC curves of various machine learning models in the internal validation set.**

Abbreviations: SVM: Support Vector Machine; XGBoost: eXtreme Gradient Boosting ; KNN: K-nearest Neighbors; GBM: Gradient Boosting Machine; AdaBoost: Adaptive Boosting.


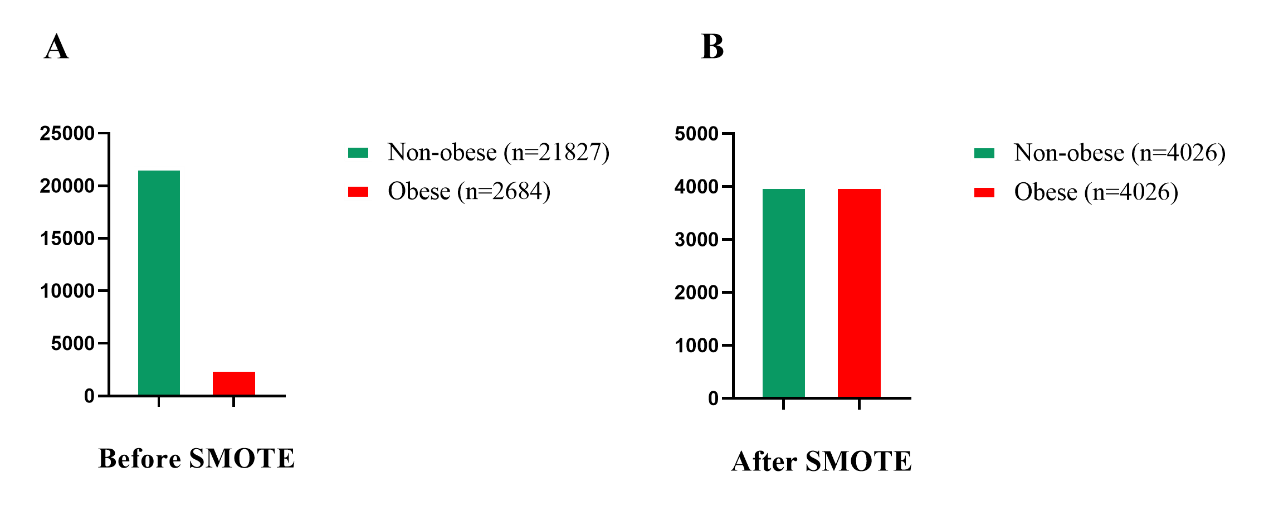


**Figure S4 Outcome distribution before and after SMOTE.**

Abbreviations: SMOTE: Synthetic Minority Oversampling Technique.


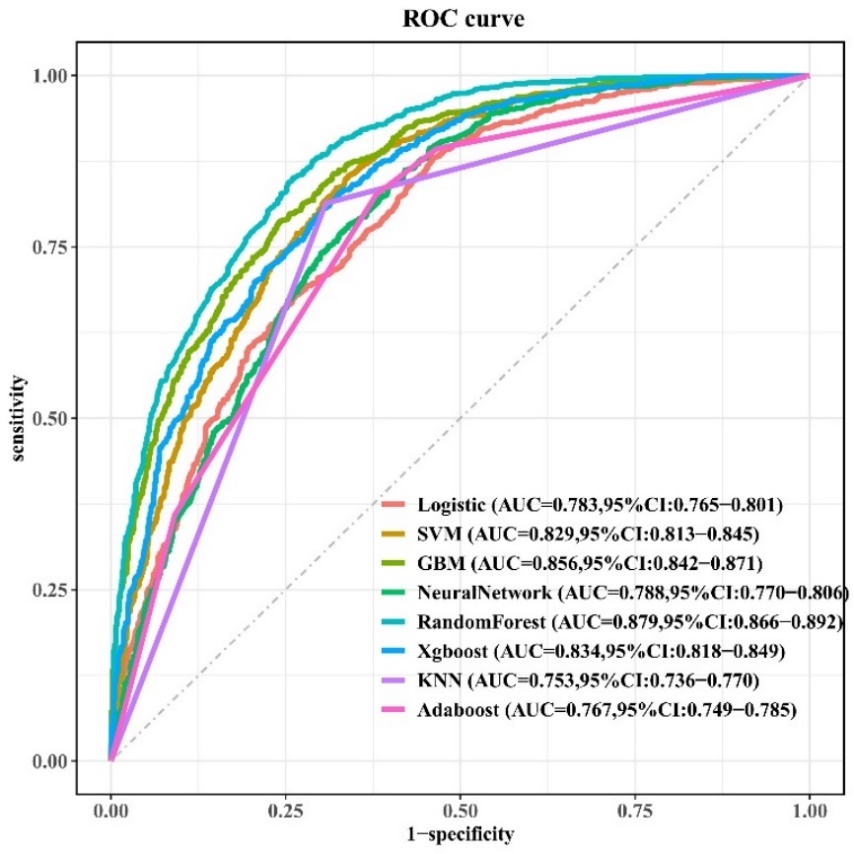


**Figure S5 ROC curves of various machine learning models trained after SMOTE in the testing set.**

Abbreviations: SVM: Support Vector Machine; XGBoost: eXtreme Gradient Boosting ; KNN: K-nearest Neighbors; GBM: Gradient Boosting Machine; AdaBoost: Adaptive Boosting.

**
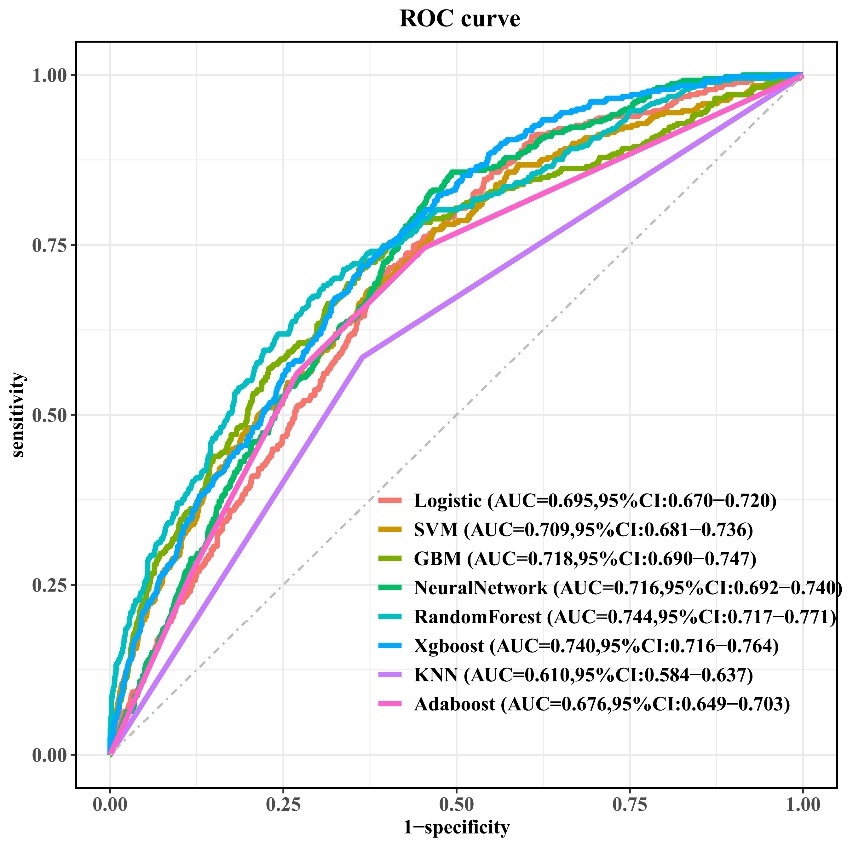
**

**Figure S6** **ROC curves of various machine learning models trained after SMOTE in the temporal validation set.**

Abbreviations: SVM: Support Vector Machine; XGBoost: eXtreme Gradient Boosting ; KNN: K-nearest Neighbors; GBM: Gradient Boosting Machine; AdaBoost: Adaptive Boosting.

**
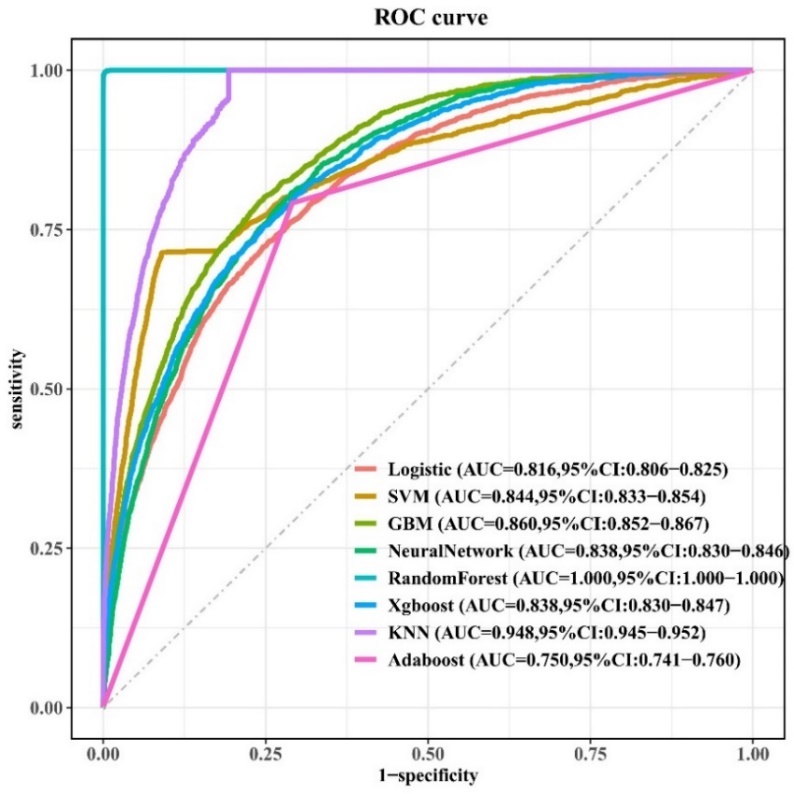
**

**Figure S7 ROC curves of various machine learning models in the complete-case training set (n-18391).**

Abbreviations: SVM: Support Vector Machine; XGBoost: eXtreme Gradient Boosting ; KNN: K-nearest Neighbors; GBM: Gradient Boosting Machine; AdaBoost: Adaptive Boosting.

**
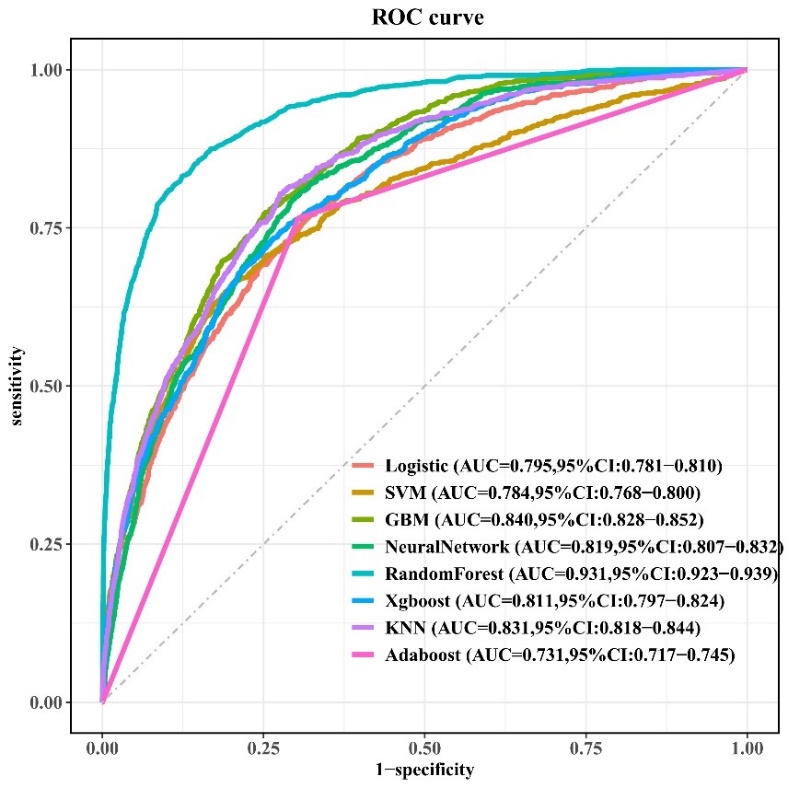
**

**Figure S8 ROC curves of various machine learning models in the complete-case testing set (n=9298).**

Abbreviations: SVM: Support Vector Machine; XGBoost: eXtreme Gradient Boosting ; KNN: K-nearest Neighbors; GBM: Gradient Boosting Machine; AdaBoost: Adaptive Boosting.

**
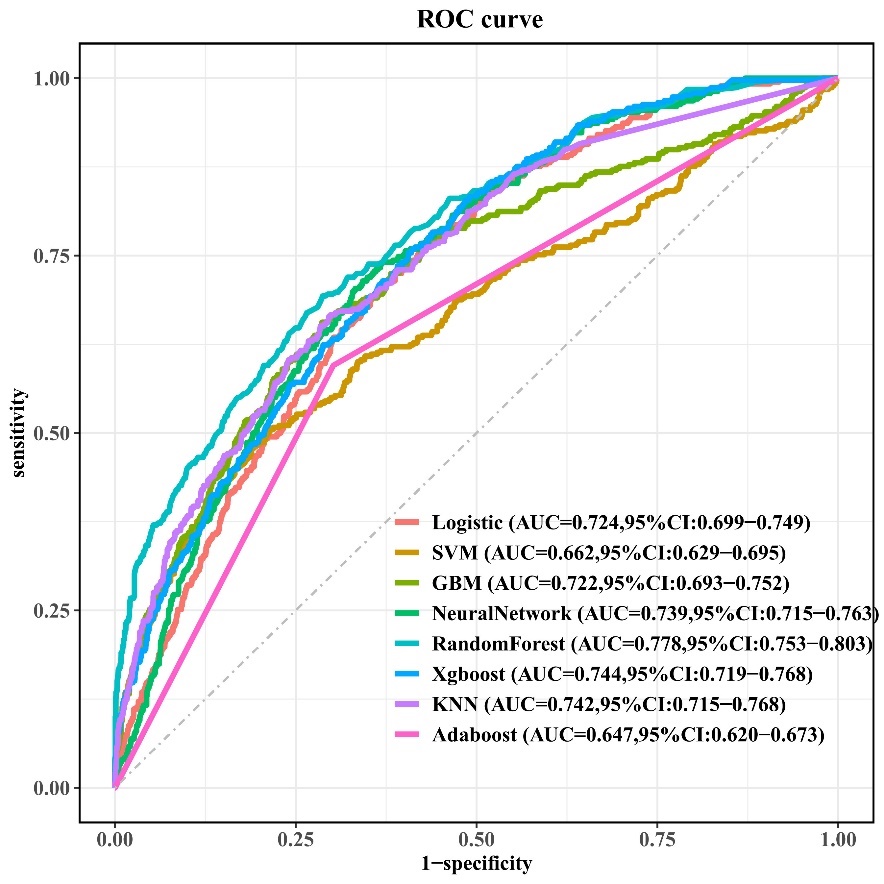
**

**Figure S9 ROC curves of various machine learning models in the complete-case temporal validation set (n=3454).**

Abbreviations: SVM: Support Vector Machine; XGBoost: eXtreme Gradient Boosting ; KNN: K-nearest Neighbors; GBM: Gradient Boosting Machine; AdaBoost: Adaptive Boosting.

**Supplementary methods**

**Behavioral variables measurement**

In the Physical Activity and Fitness in China—The Youth Study (PAFCTYS) students' physical activity (PA) was assessed using the adapted International Physical Activity Questionnaire (IPAQ)—Short Form[1]. The adapted scale utilized two items to specifically capture the frequency and duration of participation in walking, moderate (e.g., biking) and vigorous (e.g., running, heavy weightlifting) intensity physical activities that lasted for at least 10 minutes over the past seven days. The duration of various intensities of physical activity per day was calculated by dividing the total physical activity duration over seven days by 7[2]. The frequency of moderate-to-vigorous physical activity (MVPA) on weekdays or weekends was collected through the following question: “On how many days during the weekdays or weekends were you physically active for at least 60 minutes per day?” This item has shown acceptable test-retest reliability (ICC = 0.77 )[3].

The following item was used to collect muscle-strengthening exercise frequency information from the students: “In the past week, how many days did you engage in exercise to strengthen or tone the muscle, such as push-ups, sit-ups, or lifting weights?” This item was confirmed to have acceptable reliability for children and adolescent[4].

The frequency of organized sport and/or physical activity was obtained through the following question: “In the past 12 months, what forms of physical exercise have you participated?” Participants were required to indicate the type of exercise as well as the frequency, which included options of 1, 2, 3, 4, or 5 times per week. The types of activities included physical education class, morning exercise, recess activities, school sports teams, school sports clubs, extracurricular sports competitions, external sports training classes, and self-exercise[5].

Sedentary behavior was measured by 8 items adapted from the Health Behavior in School-aged Children (HBSC) survey questionnaire[6]. The following question was used: “How many hours a day do you usually do the following things in your free time?” The 8 items were: (1) Watch TV or videos or DVDs on school days; (2) Watch TV or videos or DVDs at weekends; (3) Use a computer for playing games or use console games on school days; (4) Use a computer for playing games or use console games at weekends; (5) Use a computer for chatting online, internet, emailing, homework etc. on school days; (6) Use a computer for chatting online, internet, emailing, homework etc. at weekends; (7) doing your school homework out of school hours on school days; and (8) doing your school homework out of school hours at weekends. Each question had 5 answer choices: 1= none, 2=approximately half an hour, 3=approximately 1 hour, 4=approximately 2 hours, and 5=approximately 3 hours or more[7].

Sleep duration was measured by 1 item from the Chinese version of the Pittsburgh Sleep Quality Index questionnaire, which has shown test-retest reliability (ICC =0.68) and concurrent validity (Pearson r= 0.60) for children and adolescents[8].

**Exercise intention**

Exercise intention was collected using the following question: “What is your attitude towards participating in exercise in future study and life?” The response options were as follows: 1= “I guarantee to participate in exercise every day just as now (Maintenance)”; 2=“I will consider increasing my exercise frequency, and strive to participate in exercise every day (Action)”; 3= “I will try to participate in exercise if have time, but I cannot guarantee that I will exercise every day (Preparation)”; 4= “I will try to break the habit of not exercising and be open to participate in it (Contemplation)”; and 5=“I do not like exercise, and have no plans to exercise in the future (Precontemplation).” The self-designed item of exercise intention has shown acceptable test-retest reliability (ICC= 0.52)[9].

**Body mass index (BMI)**

Student's body weight (kg) and height (cm) were recorded without shoes using a portable device (i.e., GMCS-IV; Jianmin, Beijing, China). Subsequently, BMI for each participant was calculated by dividing body weight (kg) by height (m) squared (kg/m²). The diagnosis of obesity is based on the criteria set forth by the National Health and Family Planning Commission (NHFPC) WS/T586-2018. For both boys and girls aged 6 to 17 years, overweight is defined as a BMI at or above the 85th percentile, while obesity is defined as a BMI at or above the 95th percentile, stratified by gender and age.

**Parental behavioral variables measurement**

For parents, PA and sedentary behavior were assessed using the International Physical Activity Questionnaire (IPAQ)—Short Form[1]. The method for collecting the frequency of MVPA and Muscle-strengthening exercise for parents was the same as that used for students.

**Parental BMI**

Parents were asked to self-report their most recent BMI measurement result.

**Parental attitude toward exercise**

Parental attitude toward exercise were assessed with the following question: “Do you encourage your child to participate in physical activity/exercise?” The responses to this question were measured on a 5-point scale, ranging from 1 (never) to 5 (always)[10].

**References**

1. Craig CL, Marshall AL, Sjöström M, Bauman AE, Booth ML, Ainsworth BE et al. International physical activity questionnaire: 12-country reliability and validity. Med Sci Sports Exerc. 2003;35(8):1381-1395.

2. Fan X, Cao ZB. Physical activity among Chinese school-aged children: National prevalence estimates from the 2016 Physical Activity and Fitness in China-The Youth Study. J Sport Health Sci. 2017;6(4):388-394.

3. Prochaska JJ, Sallis JF, Long B. A physical activity screening measure for use with adolescents in primary care. Arch Pediatr Adolesc Med. 2001;155(5):554-559.

4. Morrow JR, Jr., Tucker JS, Jackson AW, Martin SB, Greenleaf CA, Petrie TA. Meeting physical activity guidelines and health-related fitness in youth. Am J Prev Med. 2013;44(5):439-444.

5. Liu Y, Ke Y, Liang Y, Zhu Z, Cao Z, Zhuang J et al. Results from the China 2022 report card on physical activity for children and adolescents. J Exerc Sci Fit. 2023;21(1):1-5.

6. Liu Y, Wang M, Tynjälä J, Lv Y, Villberg J, Zhang Z et al. Test-retest reliability of selected items of Health Behaviour in School-aged Children (HBSC) survey questionnaire in Beijing, China. BMC Med Res Methodol. 2010;10:73.

7. Chen ST, Liu Y, Tremblay MS, Hong JT, Tang Y, Cao ZB et al. Meeting 24-h movement guidelines: Prevalence, correlates, and the relationships with overweight and obesity among Chinese children and adolescents. J Sport Health Sci. 2021;10(3):349-359.

8. Tsai PS, Wang SY, Wang MY, Su CT, Yang TT, Huang CJ et al. Psychometric evaluation of the Chinese version of the Pittsburgh Sleep Quality Index (CPSQI) in primary insomnia and control subjects. Qual Life Res. 2005;14(8):1943-1952.

9. Xin F, Zhu Z, Chen S, Chen H, Hu X, Ma X et al. Prevalence and correlates of meeting the muscle-strengthening exercise recommendations among Chinese children and adolescents: Results from 2019 Physical Activity and Fitness in China-The Youth Study. J Sport Health Sci. 2022;11(3):358-366.

10. Liu Y, Zhang Y, Chen S, Zhang J, Guo Z, Chen P. Associations between parental support for physical activity and moderate-to-vigorous physical activity among Chinese school children: A cross-sectional study. J Sport Health Sci. 2017;6(4):410-415.
